# GT Factor ZmGT-3b Is Associated With Regulation of Photosynthesis and Defense Response to *Fusarium graminearum* Infection in Maize Seedling

**DOI:** 10.3389/fpls.2021.724133

**Published:** 2021-11-18

**Authors:** Qianqian Zhang, Tao Zhong, Lizhu E, Mingliang Xu, Weixing Dai, Shuchang Sun, Jianrong Ye

**Affiliations:** National Maize Improvement Center, Center for Crop Functional Genomics and Molecular Breeding, College of Agronomy, China Agricultural University, Beijing, China

**Keywords:** trihelix transcription factor, ZmGT-3b, photosynthesis, defense response, growth-to-defense balance

## Abstract

It is of critical importance for plants to correctly and efficiently allocate their resources between growth and defense to optimize fitness. Transcription factors (TFs) play crucial roles in the regulation of plant growth and defense response. Trihelix TFs display multifaceted functions in plant growth, development, and responses to various biotic and abiotic stresses. In our previous investigation of maize stalk rot disease resistance mechanism, we found a trihelix TF gene, *ZmGT-3b*, which is primed for its response to *Fusarium graminearum* challenge by implementing a rapid and significant reduction of its expression to suppress seedling growth and enhance disease resistance. The disease resistance to *F. graminearum* was consistently increased and drought tolerance was improved, while seedling growth was suppressed and photosynthesis activity was significantly reduced in the *ZmGT-3b* knockdown seedlings. Thus, the seedlings finally led to show a kind of growth–defense trade-off phenotype. Moreover, photosynthesis-related genes were specifically downregulated, especially *ZmHY5*, which encodes a conserved central regulator of seedling development and light responses; ZmGT-3b was confirmed to be a novel interacting partner of ZmHY5 in yeast and in planta. Constitutive defense responses were synchronically activated in the *ZmGT-3b* knockdown seedlings as many defense-related genes were significantly upregulated, and the contents of major cell wall components, such as lignin, were increased in the *ZmGT-3b* knockdown seedlings. These suggest that ZmGT-3b is involved in the coordination of the metabolism during growth–defense trade-off by optimizing the temporal and spatial expression of photosynthesis- and defense-related genes.

## Introduction

The trihelix transcription factor (TF) family is one of the first families discovered in plants, and its family members are classified as GT factors because the GT element 5′-GGTTAA-3′ is the first *cis*-element isolated from these TFs. Trihelix family TFs can bind to different types of GT elements in promoters, such as GGTTAA, GGTAATT, GGTAAA, and GAAAAA, which are sufficient for a light-induced expression of the light-responsive genes. The DNA-binding domain of these TFs features a typical trihelix structure (helix-loop-helix-loop-helix), that is, the helices form a bundle held together by a hydrophobic core that determines their specific binding to GT elements (Kaplan-Levy et al., 2012). To date, 30 and 31 trihelix TFs have been identified in Arabidopsis (*Arabidopsis thaliana*) and rice (*Oryza sativa*), respectively. Trihelix family members are grouped into five subfamilies, GT-1, GT-2, GT, SH4, and SIP, which were named after the first identified member of each subfamily. GT-1 proteins have one trihelix DNA-binding domain, GT-2 members have two DNA-binding domains, GT-1 and GT-2 members share high sequence similarity. *Arabidopsis* GT-1 directly activates the transcription of its target genes by stabilizing the TFIIA-TBP-TATA components of the pre-initiation complex (Kaplan-Levy et al., 2012; Qin et al., 2014).

Initially, trihelix family members were found to participate in various plant developmental programs and light responses. The cloning and characterization of trihelix members from various plants have subsequently revealed their broad functional divergence in processes, including the development of floral organs, embryos, seeds, stomata, and trichomes, and biotic and abiotic stress responses (Kaplan-Levy et al., 2012; Qin et al., 2014). The biosynthesis of mixed-linkage glucan (MLG) depends on the biochemical activity of membrane-spanning cellulose-synthase-like F/H (CSLF/H). *Brachypodium distachyon* trihelix family TF BdTHX1 is involved in regulating MLG biosynthesis by controlling the transcription of *BdCSLF6* and the endotransglucosylase gene *BdXTH8* (Fan et al., 2018). Trihelix TFs are critical for plant responses to various biotic and abiotic stresses. Two *Arabidopsis* GT-1 clade members, *GT-3a* and *GT-3b*, function in the plant response to salt and pathogen stress (Park et al., 2004). AtGTL1 negatively regulates water use efficiency by modulating stomatal density; the mutation of the encoding gene increases the plant tolerance to drought stress (Yoo et al., 2010). The overexpression of the soybean (*Glycine max*) GT-2 genes, *GmGT-2A* and *GmGT-2B*, enhanced their tolerance to salt, drought, and freezing stress in transgenic *Arabidopsis* (Xie et al., 2009). BnSIP1-1 is a SIP1 member that mediates abiotic stress tolerance and abscisic acid (ABA) signaling in *Brassica napus* (Luo et al., 2017). The GT-1 subfamily member, ShCIGT, interacts with SnRK1 to mediate cold and drought tolerance in tomato (Yu et al., 2018). GTL1 is part of the MPK4-signaling cascade that coordinates pattern-triggered immunity (PTI) and effector-triggered immunity (ETI) as GTL1 positively regulates defense genes and inhibits the factors that mediate plant growth and development; *gtl1* mutants are compromised in basal immunity, PTI, and ETI (Völz et al., 2018). However, ARABIDOPSIS SH4-RELATED3 (ASR3) is phosphorylated by MPK4 to negatively regulate flg22-induced gene expression and functions as a negative regulator of PTI (Li B. et al., 2015). *OsGT*γ*-2* interacts with the GT-1 element (GAAAAA) and positively regulates rice responses to salt stress by transcriptionally regulating the expression of ion transporters, such as *OsHKT2, OsHKT1*, and *OsNHX1* (Liu et al., 2020).

Plant growth and development are constantly affected by various environmental stresses. To survive under constantly changing environmental conditions, plants must maintain a dynamic growth–defense balance to allow the optimal allocation of resources, which demands prioritization toward either growth or defense, depending on external and internal signals (Huot et al., 2014). The transcriptional regulation of gene expression is central to both plant development and responses to environmental stimuli. The induction of plant immune responses involves rapid transcriptional reprogramming that prioritizes defense- over growth-related cellular functions, which usually compromises vegetative tissue growth and yield (Alves et al., 2014). Transcriptional regulators are the key components or master regulators of the different signal transduction pathways that function during plant defense responses. TF activity alters the plant transcriptome, leading to molecular, metabolic, and phenotypic changes in favor of defense responses at the expense of normal growth (Singh et al., 2002; Alves et al., 2014). Various TFs are involved in regulating these processes by binding to specific *cis*-acting elements in the promoters of their target genes to activate or inhibit their transcription. The specific binding domain of each TF family binds to DNA *cis*-elements associated with responses to specific environmental stress; these are key features distinguishing one family from another (VanVerk et al., 2009; Mizoi et al., 2012). The best-known major TF families involved in plant stress responses are WRKYGQK (WRKY), basic leucine zipper (bZIP), myelocytomatosis (MYC), myeloblastosis (MYB), APETALA2/ETHYLENE-RESPONSIVE FACTORS (AP2/ERF), and NAM, ATAF, and CUC (NAC) TFs (Alves et al., 2014).

The MYB TF family is one of the largest plant TF families. MYBs not only have multifaceted roles in plant growth and development but also in many physiological and biochemical processes, especially the regulation of primary and secondary metabolism and responses to various biotic and abiotic stresses. MYBs may function in the cross talk linking abiotic stress responses with lignin biosynthesis pathways (Baldoni et al., 2015). Lignin, a key secondary metabolite in plants, is a major structural component of the vascular cell wall, facilitates water transport, and provides a defensive physical barrier against pathogens in various plant species. Defense-induced lignification is a conserved basal defense mechanism in the plant immune response that is used as a biochemical marker of an activated immune response. The NAC-MYB-based gene regulatory network (NAC-MYB-GRN) regulates lignin biosynthesis (Liu et al., 2018; Ohtani and Demura, 2019).

Although many recent studies have shed light on various aspects of the growth–defense trade-off, much remains to be learned about how TFs help coordinate plant growth with the appropriate responses to dynamic environmental conditions. In the current study, knocking down *ZmGT-3b* (encoding a GT-1 subfamily member) in young maize seedlings led to retarded growth, enhanced resistance to *Fusarium graminearum* infection, and enhanced drought tolerance. ZmGT-3b positively regulates the expression of genes associated with photosynthesis (especially the critical seedling growth and light response regulator *ZmHY5*) and negatively regulates the genes involved in plant defense responses. This report shows that both photosynthesis- and defense-related gene expression is simultaneously regulated by the GT TF ZmGT-3b. This TF is involved in the calibration of plant growth–defense balance to coordinate the metabolism during growth–defense trade-off by optimizing the temporal and spatial expression of photosynthesis- and defense-related genes. ZmGT-3b might serve as a molecular hub connecting developmental/environmental signaling and secondary metabolite biosynthesis by repressing or activating specific pathways.

## Materials and Methods

### Plant Growth

The length of the 7 days after germination (DAG) young seedling primary roots cultured with paper rolling was measured and used for a comparison of the seedling root growth rate, and the shoot growth rate was measured by using the soil-growth young seedlings at 12 or 15 DAG. The seedlings were cultured in controlled growth room conditions of 28/22°C (day/night) at a light intensity of 500 μmolm^−2^s^−1^ (16-h-light/8-h-night) and 40–50% relative humidity.

### *F. graminearum* Inoculation and Disease Severity Scoring

The fungal pathogen *F. graminearum* preparation and inoculation with *F. graminearum* in the field were done according to Yang et al. (2010); young seedling inoculation on primary roots was done according to Ye et al. (2013); and disease severity scoring was done according to Ye et al. (2018). Three replicates were set for each genotype with about 25 plants per replicate. The primary roots with typical symptoms were scored 48 h after inoculation (hai).

### Generation of the Transgenic Knockdown Lines of *ZmGT-3b*

We obtained a complementary DNA (cDNA) fragment encoding the c-terminal 149aa of ZmGT-3b according to the expressed sequence tag (EST; NM_001156662) and the sequence information annotated in the maize genome sequence RefGen V3.22 in 2013 by RT-PCR, to prepare a *ZmGT-3b* overexpression construct under the control of the maize *Ubiquitin* promoter, a *pBXCUN*-derived binary vector was used to generate *pUbi::cZmGT-3b* (for primer sequences, see Supplementary Table 2, and the sequence structure and primer location information, see Supplementary Table 3). The construct was transformed into *Agrobacterium* strain *EHA105* and then into the immature embryos of the maize receptor, the *Zea mays* L. variety LH244, which is a more elite inbred line for maize functional genomics research than the standard hybrid Hi-II variety, recently released from PVP by Bayer (Heidi Kaeppler, University of Wisconsin-Madison Transformation of maize; https://cropinnovation.cals.wisc.edu/maize-zea-mays/). LH244 was used as the control, CK, in the afterward experiments. Six independent transgenic events of the construct were created and selected as single-copy transfer DNA (T-DNA) integration events that expressed the transgene by the Center for Crop Functional Genomics and Molecular Breeding of China Agricultural University. These transgenic maize events were advanced, and enough T_4_ homogenous progenies were used for the following phenotypic and molecular characterizations.

### Plasmid Construction and Subcellular Localization Analysis

The full coding sequence (cds) of *ZmGT-3b* and *ZmHY5* was obtained by RT-PCR with gene-specific primers (designed according to the B73 reference genome RefGen V4.32 in 2018) amplified with reverse-transcription cDNA templates from maize young seedlings at 7 DAG. Subsequently, the sequenced clone was used for constructing BD-GT-3b, AD-HY5, and the *p1300-35S*: *ZmGT-3b-GFP* vector, with a *pCAMBIA1300*-derived binary vector by the introduction of the cds to be fused to a green fluorescent protein (GFP) driven by the Camv35S promoter (primer sequences, see Supplementary Table 2). *Agrobacterium* strain *EHA105* containing a *p1300-35S*: *ZmGT-3b-GFP* vector was cultured at 28°C overnight. Bacteria cells were harvested by centrifugation and resuspended with a buffer (10 mM MES, pH 5.7, 10 mM MgCl2, and 200 mM acetosyringone) at OD_600_ = 0.6. The leaves of 5-week-old soil-grown *Nicotiana benthamiana* were infiltrated with *Agrobacterium* cultures carrying a binary vector *p1300-35S*:*:GFP* or *p1300-35S*::*ZmGT-3b-GFP* expressing GFP or ZmGT-3b-GFP. The plants were incubated under 16-h light/8-h dark at 25°C in growth chambers. GFP fluorescence signals were detected 2 days post-infiltration (dpi). Identical *Agrobacterium* cultures that carry the binary vectors *p1300-35S*:*:GFP* or *p1300-35S*::*ZmGT-3b-GFP* expressing GFP or ZmGT-3b-GFP were also used to transform onion epidermal cells. Fluorescence images were examined and taken with LSM 880 confocal laser microscope systems and were processed using the LSM microscope imaging software. The excitation laser of 488 nm was used for imaging GFP signals.

### Maize Seedling Drought Stress Analysis

After germination, the seedlings (CK and *GT-KD*, all the four lines were tested for more than 10 times of drought treatment) were cultured in controlled growth room conditions of 28/22°C (day/night) under a light intensity of 500 μmolm^−2^s^−1^ (16-h-light/8-h-night) and 40–50% relative humidity. They were grown under well-watered conditions by maintaining soil water content close to field capacity for approximately 10 days until drought treatment. Drought stress (the cessation of watering) was imposed on the growing seedlings after the two leaves; that is, in 10 days after sowing by withdrawing water supply and keeping the plants under observation for the following 15 days, when the indications of severe withering symptom (all the leaves turned soft, drooping, and dried) were visible in almost all of the CK seedlings, then the seedlings were rewatered for 6 days. The measurements of the survived seedlings were made on day 6 following the start of rewatering when the survived seedlings were recovered. For each treatment, the normally irrigated plants were used as controls. The phenotypes and physiological indexes of the seedlings were detected, and the number of the survived seedlings and the total seedling number to obtain the survival rate (SR) were checked. The water loss rate (WLR) test was done with the third leaf of well-grown seedlings at 15 DAG; the leaves were collected and put onto big flat pallets, separately and individually under the same environment as the seedlings were growing. The weight of the five leaves was measured as a group at a given time; the weight of the lost water was obtained by subtracting this weight from the fresh weight, and the WLR was calculated as a percentage of the weight of the lost water to the initial fresh weight of the given group. Data are means ± SD of all three replicates.

### Analysis of Cell Wall Components, Cellulose, and Lignin Content

Twelve-day-old (12 DAG) *GT-KD* and CK maize seedlings were harvested, dried, and homogenized to a fine powder using a mixer mill (MM400, Retsch Technology, Haan, Germany) at 25 Hz for 2 min. About 100 mg of the powdered seedling tissue was sequentially ultrasonicated for 15 min by mixing two times with 1 ml of methanol, two times with phosphate buffered saline pH (7.0) containing 0.1% (v/v) Tween 20, two times with 1 ml of 95% ethanol, two times with 1 ml of (1:1) chloroform: methanol, and two times with 1 ml of acetone. The samples were centrifuged at 16,000 g for 10 min, and the pellets were dried at 50°C. The remaining cell wall extract was used for the determination of total lignin content. The lignin content of seedlings was quantified using the acetyl bromide soluble lignin method. The seedling tissue was macerated in 72% (v/v) sulfuric acid for 2 h, diluted with 112 ml deionized water, and thereafter autoclaved at 121°C for 1 h. The acid-insoluble lignin (AIL) was quantified using pre-weighed medium coarse-ness glass crucibles, while a UV/VIS spectrometer was used to determine the acid-soluble lignin (ASL) content at 205 nm with an ultraviolet spectrophotometer (TU-1901). To hydrolyze the cell wall polysaccharides, 10 mg of the destarched sample was mixed with 200 μl 72% sulfuric acid and incubated at 60°C for 1 h, and then the sulfuric acid was diluted to 3% with distilled water for hydrolysis at 121°C for 1 h. After cooling to room temperature, the supernatant was collected, erythritol was added as an external standard and was then neutralized with barium carbonate. The sugars in the supernatant were separated by using an SP0810 column (Shodex) on the UHPLC system (Agilent-1260). The content of the detected sugars was calculated based on the standard curves of glucose, xylose, mannose, galactose, and arabinose. Error bars indicate the SD of three biological replicates.

### Leaf Chlorophyll Content, Net Photosynthetic Rate, and Transpiration Rate Analysis

Leaf chlorophyll content was measured from the recently expanded leaves of the CK and *GT-KD* seedlings grew at 12 or 15 DAG, with a SPAD meter (SPAD-502 Plus, Konica Minolta, Inc., Tokyo, Japan) under a saturating actinic light (660 nm) with an intensity of 1,100 μmol m^−2^s^−1^. The middle widest part of the recently expanded leaf of every seedling, that is the second leaf of the 12 DAG seedlings and the third leaf of the 15 DAG seedlings, was used for the SPAD value measurement. The net photosynthetic rate (Pn) and transpiration rate (TR) were measured from the latest expanded leaf (the third leaf) of the 15 DAG seedlings (with three leaves and a heart leaf) with a portable LI-6400XT Portable Photosynthesis System (LI-COR, Lincoln, NE, USA), recorded at a saturating actinic light (660 nm) with an intensity of 1,100 μmol m^−2^s^−1^, at the time from 09:00 to 12:00 in the morning. All the measurements were conducted on the middle part of the latest expanded leaves following the instructions of the manufacturer. Five replicates were randomly taken for each genotype.

### RNA Extraction and Transcriptome Sequencing

To compare the transcriptomes between *GT-KD* (three different lines, G3, G4, and G6, were used) and CK (Control, LH244) with or without inoculation, the inoculated (the seedlings were inoculated at 5 DAG and sampled at 18 hai) and the non-inoculated *GT-KD* and CK seedling samples (whole seedlings without the kernel) were collected at the same time and used for RNA extraction and deep sequencing. Three micrograms of total RNA from each sample were used for transcriptome sequencing at Novogene (http://www.novogene.com/). Sequencing was performed on each library to generate 100-bp paired-end reads employing the high-throughput sequencing platform highseq3000. Read quality was checked using FastQC, and low quality reads were trimmed using Trimmomatic version 0.32 (http://www.usadellab.org/cms/?page=trimmomatic). The clean reads were aligned to the masked maize genome database for the mapping, calculation, and normalization of gene expression (the updated *Z. mays* B73 reference genome AGPv4, http://ensembl.gramene.org/Zea_mays/Info/Index). Differentially expressed genes (DEGs) were defined with the *p*-value of 0.05, with statistical correction using the Benjamini–Hochberg false discovery rate (FDR) of 0.05 in cuffdiff. The parameters used for screening DEGs were the fold change (FC) of the expression level (FC ≥ 2 or FC ≤ 0.5 under the value of *p* ≤ 0.05 and FDR ≤ 0.05), compared with the expression level in the control transcriptome. Defining DEG and cluster analysis was done using the software Cluster 3.0. For the GO term analysis, significantly DEGs were analyzed using AGRIGO (http://bioinfo.cau.edu.cn/agriGO/) between the tested conditions (Trapnell et al., 2010).

### Quantitative Reverse-Transcription PCR Analysis

Real-time PCR (RT-qPCR) was used to check the relative expression level and validate the RNA-sequencing (RNA-seq) results for those light- and defense-related genes that showed different FCs in the expression level between the *GT-KD* and CK seedlings. Primers were designed using the Primer Express software, according to standard parameters for real-time RT-qPCR assays (Bio-Rad Laboratories, Hercules, CA, USA). For quantitative reverse-transcription PCR **(**qRT-PCR) experiments, total RNA was extracted from young seedling tissues (the CK and different *GT-KD* lines from three different transgenic events were used) at 6 DAG with RNAiso Plus (Takara Bio, Shiga, Japan) according to the user manual. First-strand cDNA was synthesized by using about 1 μg of total RNA and RT Master Mix with gDNA Remover (Takara Bio, Shiga, Japan), which contains M-MLV reverse transcriptase, oligo (dT) primer. qRT-PCR analyses were conducted using PowerUp™ SYBR Green Master Mix (Applied Biosystems, Carlsbad, CA, USA) on an ABI 7500 thermocycler (Applied Biosystems, Carlsbad, CA, USA). The primer pair GAPDH-qF/qR was used to monitor *GAPDH2* expression as an internal control. The sequences of all the primers for qRT-PCR analysis are given in Supplementary Table 2. The relative expression levels were calculated using the relative quantization according to the quantification method (2^−Δ*ΔCt*^) (Livak and Schmittgen, 2001) and plotted with standard errors. A variation in expression was estimated using the three biological replicates independently by comparative qRT-PCR.

### Yeast Two-Hybridization Assay

A bait construct BD-GT-3b (pGBKT7-GT-3b) was generated by cloning the *ZmGT-3b* coding sequence with the primers pGBKT7-GTF/pGBKT7-GTR (Supplementary Table 2) and ligated into *Eco*RI/*Bam*HI-digested pGBKT7. The *ZmHY5* coding region was cloned and ligated into *Eco*RI/*Bam*HI-digested pGADT7 as a prey construct, AD-HY5. After confirmation by sequencing, a pair of bait and prey vectors were co-transformed into the yeast AH109 strain according to the Matchmaker Gold Yeast Two-Hybrid System User Manual (Clontech, Palo Alto, CA, USA). Yeast colonies growing on an SD-Trp/Leu plate were diluted to SD-Trp/Leu/His plate with X-α-gal to further determine the binding ability. The BD-GT-3b and pGADT7 pair was used as a negative control.

### Luciferase Complementation Image Assay

For the bimolecular fluorescence complementation (BiFC) assay, the coding sequence of ZmGT-3b and ZmHY5 was cloned into JW771 (N-terminal half of luciferase, NLUC) and JW772 (C-terminal half of luciferase, CLUC) using the ClonExpress II One Step Cloning kit (Vazyme Biotech, Nanjing, China), to produce NLUC-GT-3b and CLUC-HY5. These constructs were transformed into *Agrobacterium tumefaciens* (strain GV3101), after which, the *Agrobacterium* cells were cultured to OD600 = 0.8, pelleted, and suspended in a buffer (10 mM methylester sulfonate, 10 mM MgCl_2_, and 150 mM acetosyringone, pH 5.7). The suspended cells were incubated at room temperature for ~3 h and infiltrated into 5-week-old *N. benthamiana* leaves in different combinations using a needleless syringe. The infiltrated plants were placed at 28°C for 48 h and then injected with 1 mmol/L beetle luciferase (Beetle luciferin, Promega, Madison, WI, USA) at the initial injection site, and then the fluorescence signal was imaged using the Tanon-5200 imaging system. These experiments were repeated, and each combination was infiltrated with multiple leaves.

### Statistical Analyses

Statistical analysis was conducted using Student's *t*-test between the control (CK) and mutant *GT-KD* seedlings to determine a statistical significance in three independent experiments. All the data from experiments shown in bar graphs were shown as mean ± SD. Statistical analysis was performed using the paired Student's *t*-test for pairwise comparison. Asterisks indicate significant differences (^*^p < 0.05 for significant and ^**^p < 0.01, ^***^p < 0.001 for highly significant values). All experiments were independently repeated at least three times. All figures were generated using Adobe Illustrator CS6.

## Results

### The Trihelix TF Gene *ZmGT-3b* Responds Rapidly to *F. graminearum* Infection in Maize Seedlings

We previously identified the quantitative trait locus (QTL) *qRfg1* on chromosome 10, which explained 36.6% of the total variation in maize resistance to *F. graminearum*-induced stalk rot (Yang et al., 2010). We then inoculated two maize near-isogenic lines (NILs) carrying either the resistant *qRfg1* allele (R-NIL) or the susceptible *qRfg1* allele (S-NIL) with *F. graminearum* to evaluate the role of *qRfg1* in resistance to this pathogen (Ye et al., 2013). During the investigation of maize stalk rot disease resistance mechanism with NILs, we found that the trihelix TF gene *ZmGT-3b* (*GRMZM2G325038*) was expressed at relatively high levels in non-inoculated R-NIL-seedling roots, but its expression was significantly decreased after inoculation (Supplementary Figure 1A). Subsequently, we also detected a significant reduction in *ZmGT-3b* expression in response to *F. graminearum* inoculation in maize seedlings with or without the QTL *qRfg2* on chromosome 1 (data not shown). ZmGT-3b belongs to the GT-1 clade of the plant-specific trihelix TF family. Based on transcriptome data, *ZmGT-3b* is primarily expressed in a few kinds of young tissues, such as the primary root, ear primordium (2–8 μm), and embryo at 20 days after pollination (DAP) as well as the presheath and other tissues (Supplementary Figures 1B,C).

Consistent with its expression profile, the promoter region of *ZmGT-3b* (2,000 bp, 5′-upstream sequence of the starting codon ATG) contains 19 light-responsive *cis*-elements (LREs) and 21 defense or stress response-related *cis*-elements, including 1 W-box/Box-w1 (pathogen-inducible *cis*-element), 2 MBSs (MYB binding site involved in drought inducibility), 2 ABREs (*cis*-acting element involved in the ABA response), 8 TGACG-motifs (*cis*-acting regulatory element involved in the MeJA response), etc., (Supplementary Table 1). These elements/motifs likely allow the induction of *ZmGT-3b* in response to various development- and biotic/abiotic stress-related signals.

### *ZmGT-3b* Expression Is Light-Responsive and ZmGT-3b Localizes to the Nucleus

**Figure 1:**
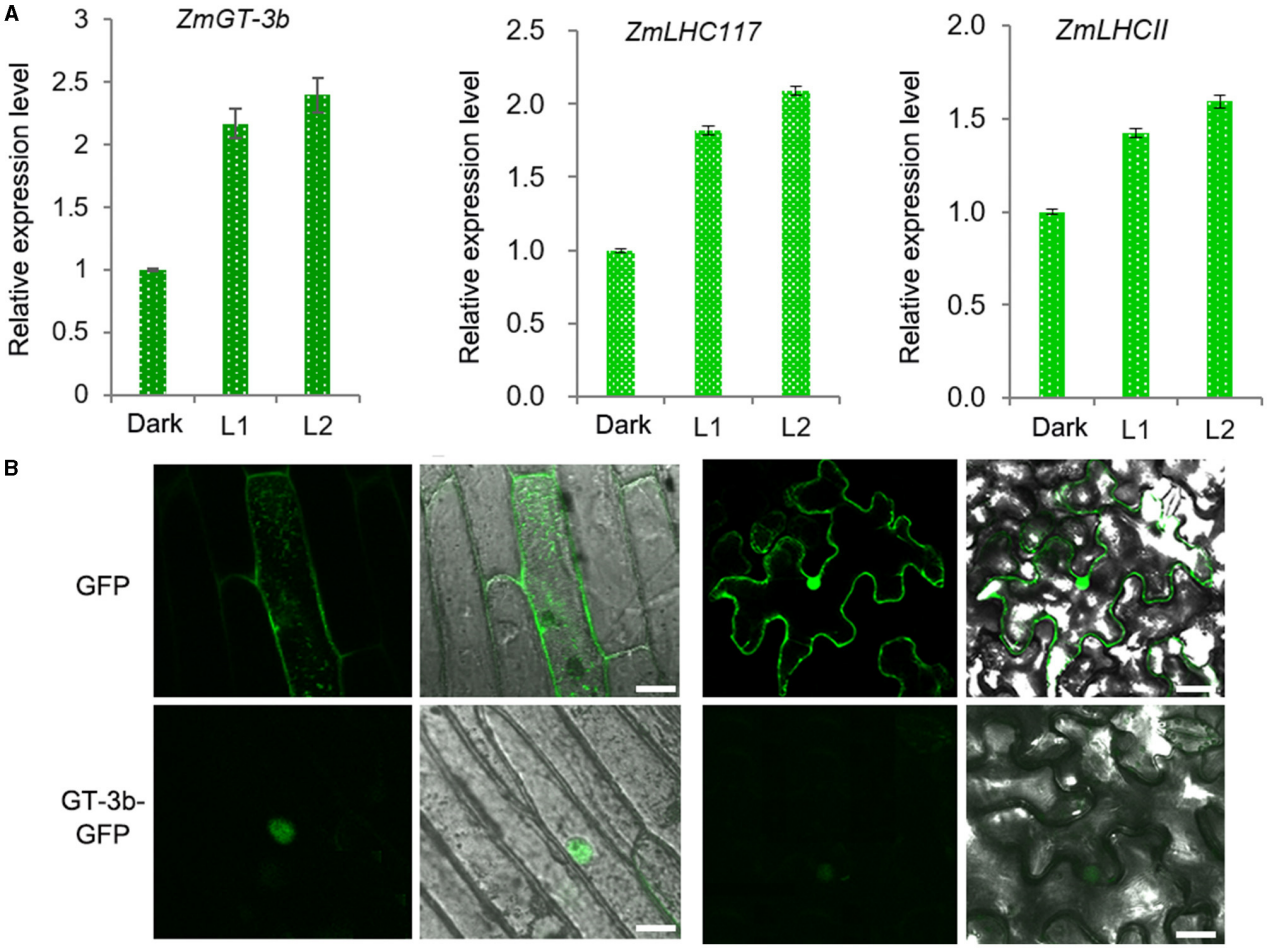
Light-responsive *ZmGT-3b* expression and subcellular localization of ZmGT-3b. **(A)** The expression of *ZmGT-3b, ZmLHC117*, and *ZmLHCII* (they are the two randomly selected typical light-responsive genes that encode the components of the light harvesting complex) is induced by light. “Dark” represents control seedlings (CK, maize inbred line LH244, wild type) that were grown in the dark for 5 days; L1 and L2 represent CK seedlings transferred to the light for 1 and 2 h after 5 days of growth in the dark, respectively. Error bars denote the mean ± SD of *n* = 3 replicates. **(B)** ZmGT-3b-GFP localizes to the nucleus in onion epidermal cells and *Nicotiana benthamiana* leaves that were infiltrated with *Agrobacterium tumefaciens* carrying *p35S*: *ZmGT-3b-GFP* and observed 48 h after infiltration. Green fluorescent protein (GFP) is the control transformed with an empty vector containing the *p35S::GFP* expression cassette in the pCAMBIA1300 backbone. The images were taken under a confocal microscope. Bar =10 μm.

In agreement with the discovery of 19 LREs in the promoter of *ZmGT-3b, ZmGT-3b* expression was rapidly induced by light in the young seedlings of the maize inbred line LH244 [wild type, used as the control (CK)], which were grown in the dark for 5 days. This expression pattern is similar to the light-responsive expression of *ZmLHC117* and *ZmLHCII*, which are the two typical light-responsive genes (selected randomly) and encode the components of the light-harvesting complex (Figure 1A). According to the B73 reference genome RefGen V4.32, *ZmGT-3b* contains 3 exons and encodes a predicted protein of 246 amino acids in length, containing the nuclear localization sequence (NLS) RKKLKRP at its N-terminus. We transferred the vector *p35S*: *ZmGT-3b-GFP*, which contained the full coding sequence of *ZmGT-3b* that was fused to the N-terminus of *GFP* under the control of the cauliflower mosaic virus (CAMV) 35S promoter, into onion epidermal cells and *N. benthamiana* leaves and analyzed the subcellular localization of ZmGT-3b-GFP. This result showed that ZmGT-3b-GFP localized specifically to the nuclei of the epidermal cells (Figure 1B).

### Knockdown of *ZmGT-3b* Diminishes Seedling Growth With Reduced Photosynthetic Activity in Maize

**Figure 2:**
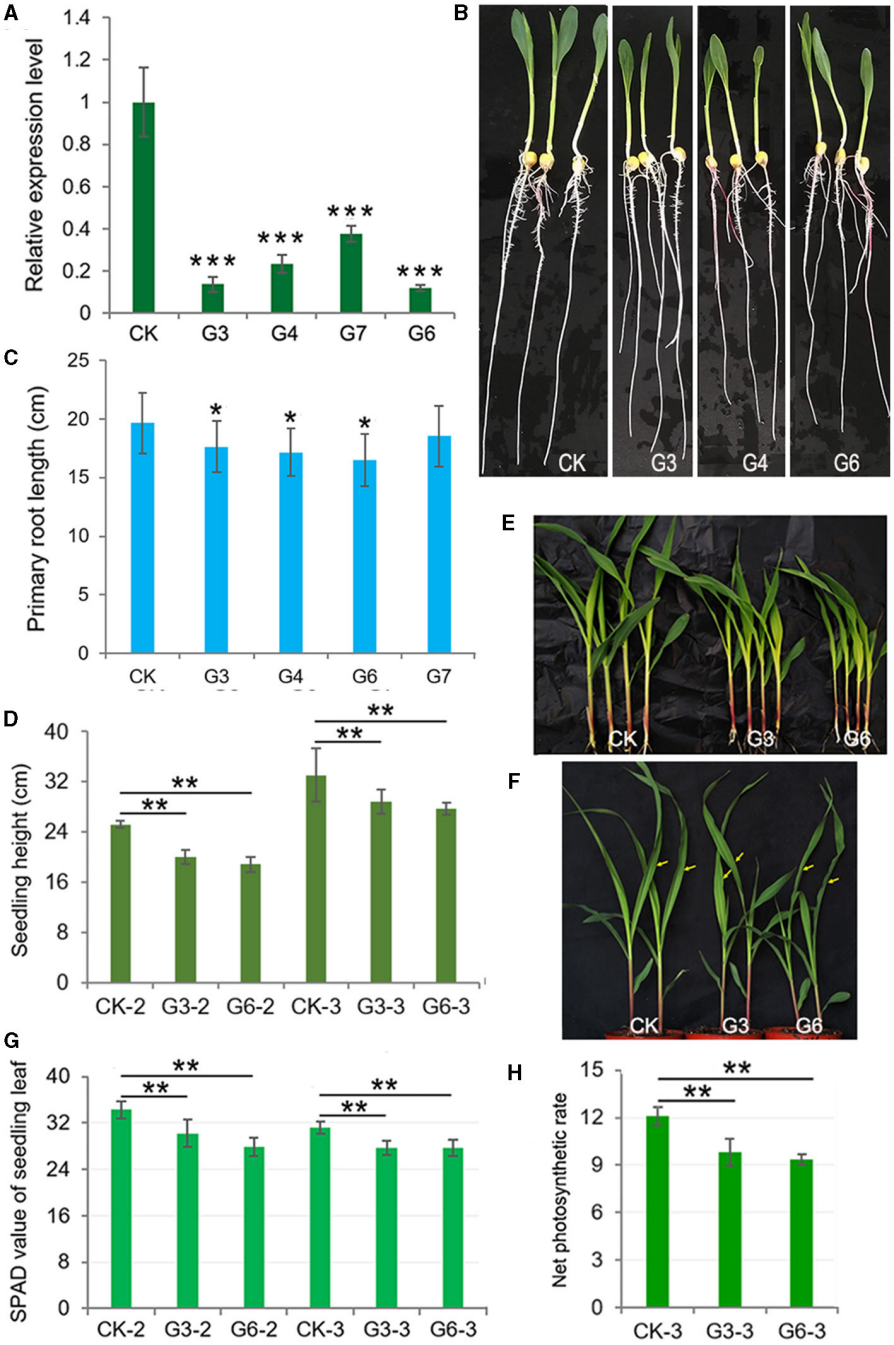
**(A)** significantly decrease in *ZmGT-3b* expression suppresses the growth of maize seedlings owing to reduced photosynthetic rates and chlorophyll contents. **(A)** *ZmGT-3b* expression in the primary roots of transgenic maize seedlings is significantly reduced, compared with CK (the maize inbred line LH244, wild type) at 7 days after germination (DAG), with a pair of primers spanning the second and the third exon (Supplementary Table 2) to detect the transcript level of the full complementary DNA (cDNA) of *ZmGT-3b*. G3, G4, G6, and G7 represent maize seedlings from four transgenic events, which were transformed with the partial coding sequence of the C-terminal of ZmGT-3b. Seedling phenotypes **(B)** and average primary root lengths **(C)** of 7 DAG maize seedlings (*n* > 15). The average seedling height [**(D)**, *n* > 15] and shoot phenotype [**(E)** for 12 DAG and **(F)** for 15 DAG] of soil-cultured maize seedlings were grown in a greenhouse under natural light. Values are the mean ± SD (*n* > 15). Twelve DAG seedlings with two leaves and a heart leaf are denoted as CK-2, G3-2, and G6-2, and 15 DAG seedlings with three leaves and a heart leaf are denoted as CK-3, G3-3, and G6-3 [used to measure the net photosynthetic rate (Pn)]. The SPAD values **(G)** of the above seedlings were obtained from the central widest part of the newest expanded leaf, that is, the second leaf of 12 DAG seedlings or the third leaf of 15 DAG seedlings. **(H)** The Pn (μmol CO_2_·m^−2^s^−1^) of the central widest part of the third leaf of 15 DAG seedlings [indicated by an arrow in **(F)**] was measured from 09:00 to 12:00 under natural light in a greenhouse. Error bars denote the mean ± SD of the biological replicates. The asterisk * indicates a statistically significant difference between CK and the *GT-KD* lines, as calculated by the paired Student's *t*-test (*p < 0.05, **p < 0.01, ***p < 0.001).

We obtained a cDNA fragment encoding the c-terminal 149aa of ZmGT-3b according to the available EST (NM_001156662) and *ZmGT-3b* sequence information annotated in the maize genome sequence RefGen V3.22 in 2013, and then prepared a construct that contains the partial cDNA of *ZmGT-3b* under the control of maize *Ubiquitin* promoter, *pUbi::cZmGT-3b*. Six independent transgenic events harboring the construct were obtained, genotyped with a forward primer originated from the *Ubiquitin* promoter sequence and a reverse primer from the transformed cDNA sequence, selfed, and their homogenous seeds were harvested for functional analysis of *ZmGT-3b* in maize. To our great surprise, *ZmGT-3b* transcript levels in the primary roots of transgenic seedlings from the four events (G3, G4, G6, and G7) were all significantly reduced, compared with CK seedlings; the relative transcript level of G6 was only 0.11, and that of G7 was 0.375 compared with CK by qRT-PCR (Figure 2A), with a pair of primers designed with an N-terminal coding sequence spanning the second and the third exon. Later, such results were further confirmed by qRT-PCR in independent RNA samples (sampled both young leaves and roots) multiple times with a different pair of primers designed with different combinations of the three exons of *ZmGT-3b* or with the transformed cDNA sequence. These results indicated that *ZmGT-3b* transcript levels are significantly decreased in both the leaves and roots of the transgenic maize seedlings (Supplementary Figure 2A). Also, these results indicate that the transformation induced *ZmGT-3b* transcript levels (both the endogenous and the transformed sequence of *ZmGT-3b*) to be significantly decreased in the maize seedling. We named the *ZmGT-3b* mutants with a significant reduction in *ZmGT-3b* transcript levels as *GT-KD*.

The average primary root length of CK seedlings was ~19.66 cm at 7 DAG, the average primary root length of *GT-KD* seedlings was reduced by ~10.25, ~16.17, and ~5.72% for G3, G6, and G7, respectively (Figures 2B,C). The average seedling height was also significantly lower in G3 and G6 than in CK seedlings at both 12 and 15 DAG when grown in a greenhouse under a 16-h-light/8-h-dark cycle, with reductions of ~18.06 and ~19.13% for G3 and G6 at 12 DAG, respectively (Figures 2D,E). When plants were grown in the field, plant height and 100-kernel-weight (HKW) of mature *GT-KD* plants did not significantly differ from those of CK plants grown in the same field (Supplementary Figures 2B,C). The retarded growth of *GT-KD* seedlings induced by the severe knockdown of *ZmGT-3b* suggests that *ZmGT-3b* is involved in the positive regulation of maize seedlings' root and shoot growth but has little effect on mature plant growth.

As trihelix TFs were initially found to participate in the light response, we examined the chlorophyll content and Pn of *GT-KD* seedlings. The SPAD value is commonly used to estimate chlorophyll content. The average SPAD value of 12 DAG CK seedlings was 34.3, whereas the average SPAD value of 12 DAG *GT-KD* seedlings decreased by ~11.95 and ~18.6% in G3 and G6, respectively, compared with CK. In 15 DAG seedlings, the average SPAD value decreased by ~10.9 and ~11.2% in G3 and G6, respectively, compared with CK. We also measured the Pn of the newest expanded leaf of each 15 DAG seedling. The Pn value ranged from 11.3 to 12.7 μmol CO_2_·m^−2^s^−1^ in CK and from 8.9 to 11.2 μmol CO_2_·m^−2^s^−1^ in *GT-KD* seedlings. The average Pn decreased by ~18.9 and ~22.8% in G3 and G6, respectively, compared with CK seedlings (Figures 2F–H). These findings suggest that ZmGT-3b is involved in the positive regulation of the young seedling root and shoot growth by regulating chlorophyll biosynthesis and photosynthetic activity.

### *ZmGT-3b* Knockdown Improved Disease Resistance and Drought Tolerance in Maize Seedlings

**Figure 3:**
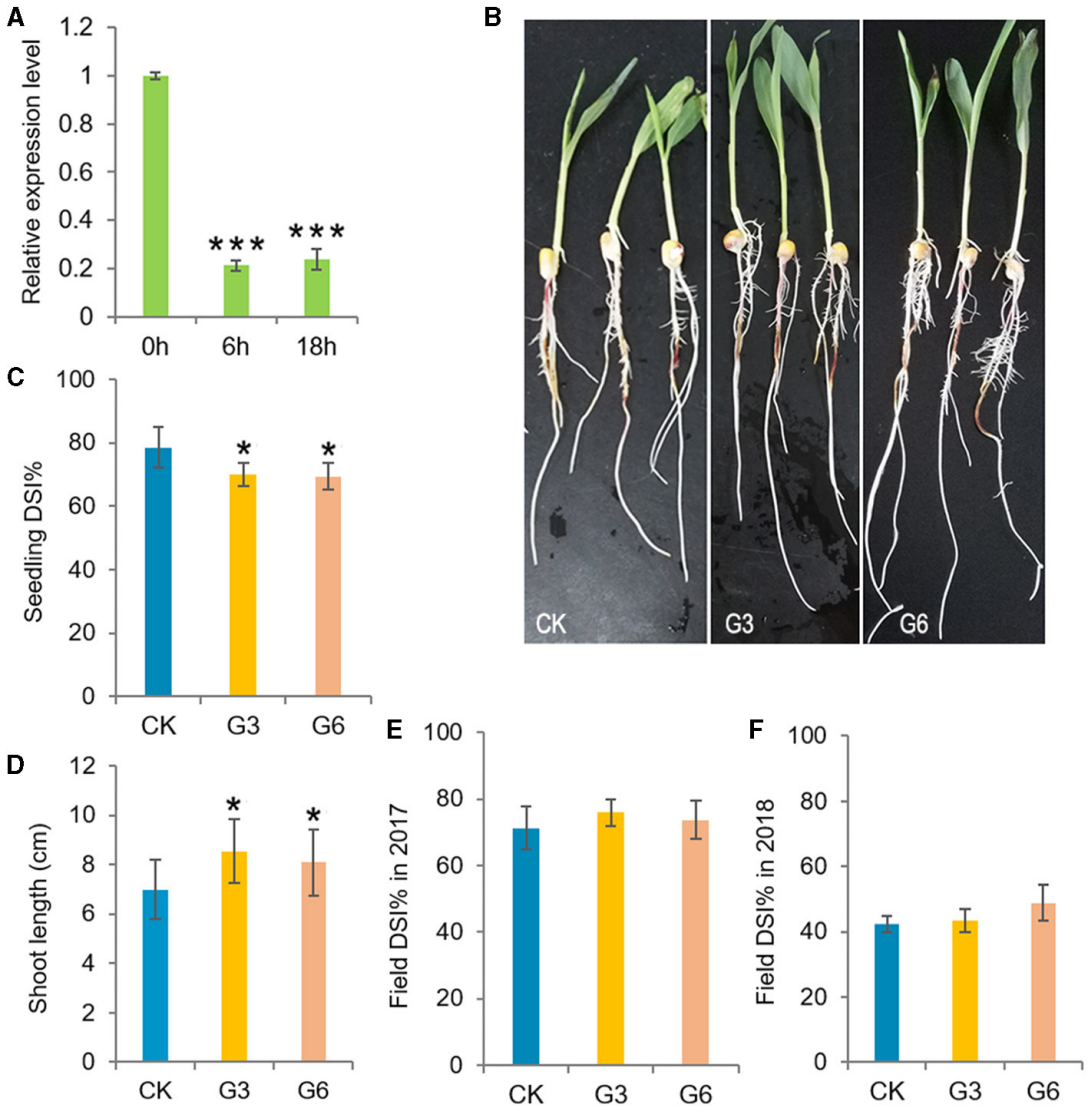
Analysis of the disease resistance of transgenic maize with a significant reduction in *ZmGT-3b* expression. **(A)** *ZmGT-3b* expression in young CK (maize inbred line LH244, wild type) seedlings significantly decreased after *F. graminearum* inoculation. “0,” “6,” and “18 h” indicate hours after the inoculation of seedlings at 5 DAG. Both shoot and root growth **(B)** were much better in the inoculated *GT-KD* seedlings compared with the inoculated CK seedlings with similar levels of disease severity, the disease severity index (DSI, *n* > 20 individual seedlings) values of G3 and G6 seedlings were also significantly lower than that of CK seedlings **(C)**, and the shoot length of the diseased seedlings of G3 and G6 were longer than that of CK [**(D)**, *n* > 20 individual seedlings]. **(E,F)** The DSI of mature transgenic maize (*Zea mays*) plants was similar to (or higher than) that of CK plants in field inoculation experiments. The data of DSI are mean ± SD (*n* > 25 individual plants). The asterisk indicates a statistically significant difference between CK and the *GT-KD* lines, as calculated by a paired Student's *t*-test (*p < 0.05, ***p < 0.001).

Plant growth and immune responses both consume large amounts of energy. The deployment of defense mechanisms is crucial for plant survival, thus the allocation of energy to defense activation generally comes at the expense of plant growth due to limited resources (Huot et al., 2014). Consistently, *ZmGT-3b* expression was significantly decreased in young CK seedlings after *F. graminearum* inoculation (Figure 3A). *ZmGT-3b* knockdown led to retarded growth in young seedlings (Figures 2A–E) but improved the resistance to *F. graminearum* infection. The disease severity index (DSI) of the inoculated G3 and G6 seedlings was markedly lower than that of the inoculated CK seedlings, both the shoot and root growth phenotypes of *GT-KD* seedlings were much better than CK seedlings following inoculation, the shoot length of *GT-KD* seedlings at 48 hai was significantly longer than that of CK seedlings (Figures 3B–D). However, following the field inoculation of mature maize plants, the DSI of *GT-KD* plants was similar to (or higher than) that of CK plants (Figures 3E,F). Taken together, the finding that *ZmGT-3b* is only highly expressed in a few young tissues (Supplementary Figures 1B,C), and that young *GT-KD* seedlings show retarded growth and improved disease resistance, suggesting that *ZmGT-3b* is a positive regulator of growth and a negative regulator of disease resistance in maize seedlings.

**Figure 4:**
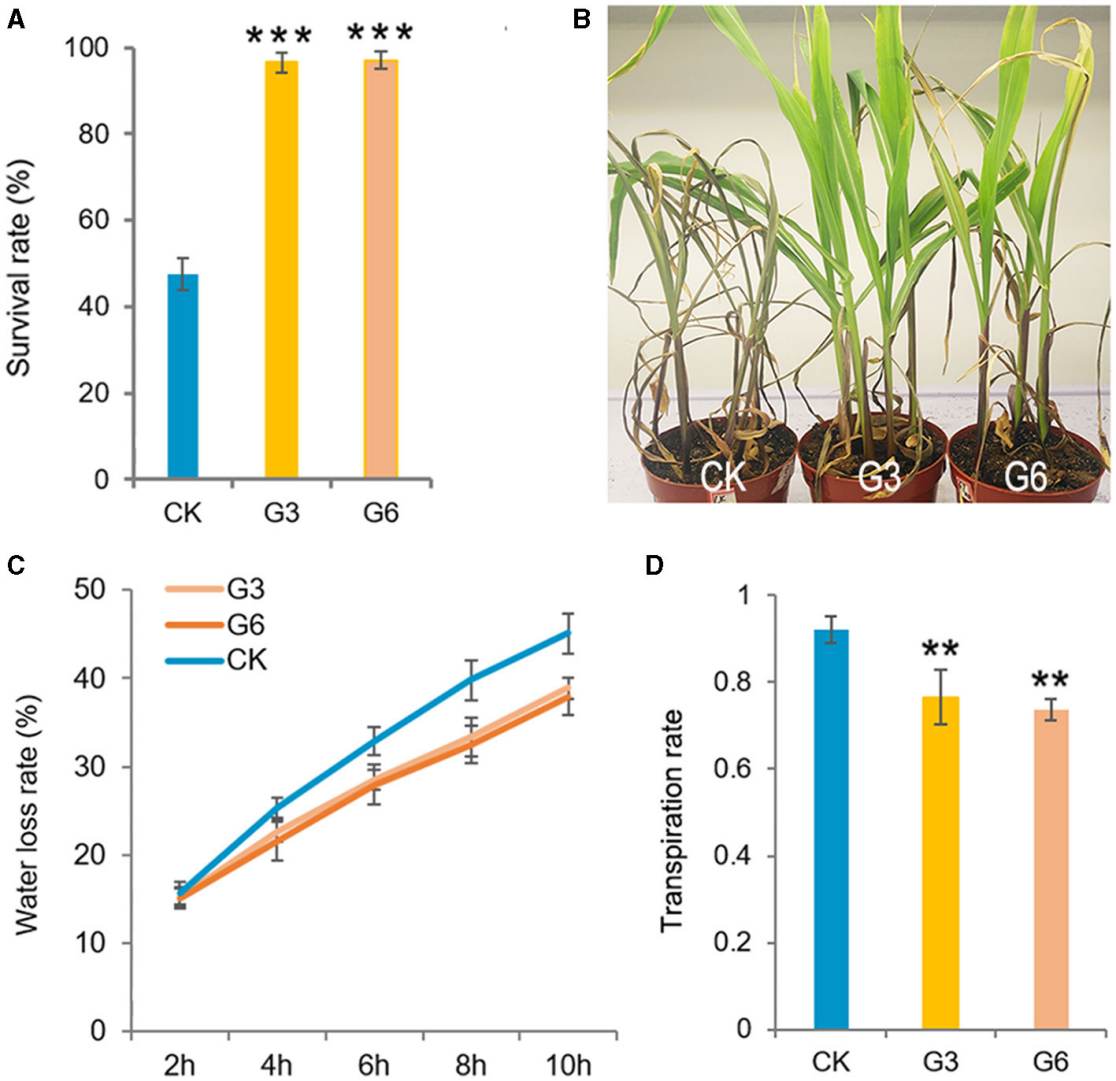
*ZmGT-3b* knockdown increases drought tolerance in *GT-KD* seedlings. The survival rate (SR) **(A)** and the phenotypes of the wilted or survived maize seedlings rewatered after serious drought treatment **(B)**. CK (maize inbred line LH244, wild type), G3 and G6 seedlings were shown at 6 days after rewatering. Water was withheld from growing seedlings at the two-leaf stage, and the plants were rewatered when all CK seedlings were severely wilted (at ~25 DAG). Values are the mean ± SD (*n* > 25) **(C)** Leaf water loss rates (WLRs) of young maize (*Z. mays*) seedlings. The leaf WLRs are shown as the means of the percentage of leaf water loss ± SD (*n* = 6). Three independent experiments were performed with the third leaf of each seedling. **(D)** The transpiration rate (TR) of the maize seedling leaves. The estimated TR was obtained from the central widest part of the third leaf, which is the newest expanded leaf of 15 DAG CK or *GT-KD* seedlings (with three leaves and a heartleaf). The TR value was measured simultaneously with the Pn from 09:00 to 12:00 under natural light in a greenhouse, TR values are the mean ± SD (*n* = 5). The asterisks indicate a statistically significant difference between CK and the *GT-KD* lines, as calculated by a paired Student's *t*-test (**p < 0.01, ***p < 0.001).

Unexpectedly, when we stopped watering the growing seedlings after the two-leaf stage, all CK seedlings severely wilted at ~25 DAG, and *GT-KD* seedlings wilted less and would return into life quickly after being rewatered (Figure 4B), indicating that *GT-KD* seedlings were drought tolerant. We then tested the leaf WLR and SR of maize seedlings. All the tested four transgenic lines showed similar drought tolerance with similar SR. To be consistent with previous experiments, the obtained data of G3 and G6 lines were shown here. Compared with CK, the leaf WLR and SR of both G3 and G6 lines were significantly lower than those of CK (Figures 4A–C). We further estimated the transpiration rate (TR) of the newest expanded leaf of each 15 DAG seedling. The estimated TR range of CK seedlings was 0.884–0.96 μmol H_2_O m^−2^s^−1^, whereas that of *GT-KD* seedlings was 0.664–0.818 μmol H_2_O m^−2^s^−1^. The average TR of G3 and G6 seedlings was ~22.12 and ~25.73% less than that of CK seedlings, respectively (Figure 4D). However, there was no difference in stomata density and aperture between *GT-KD* and CK seedling leaves (data not shown). These indicate that *GT-KD* seedlings with the knocked-down expression of *ZmGT-3b* are more drought tolerant than CK seedlings.

### Photosynthesis-Related Genes Are Significantly Downregulated in the *ZmGT-3b* Knockdown Lines

**Figure 5:**
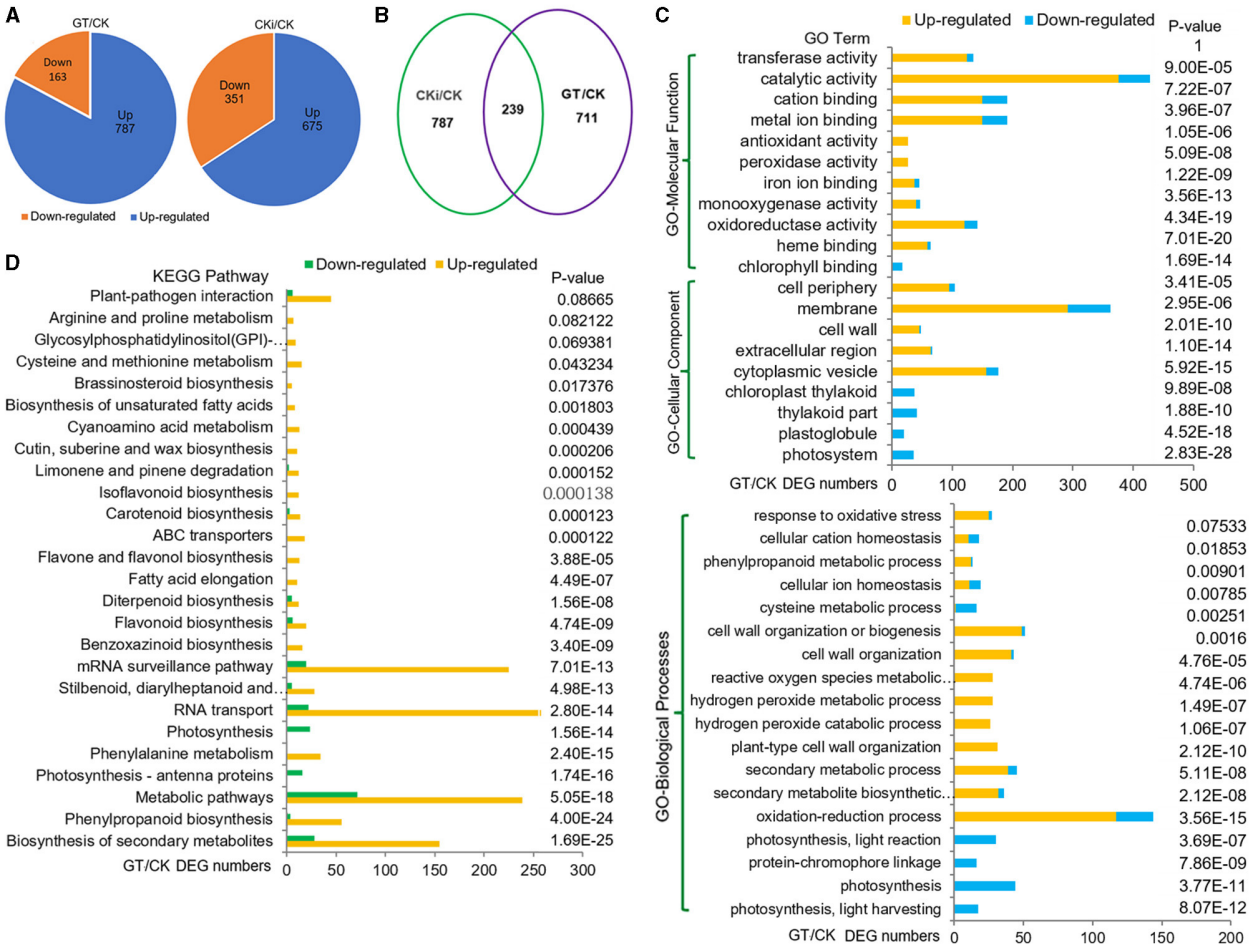
Transcriptome reprogramming induced by the knockdown of *ZmGT-3b*. **(A)** Compared with CK (maize inbred line LH244, wild type), *ZmGT-3b* knockdown induced a differential expression of 950 genes (GT/CK), while the inoculation with *F. graminearum* induced a differential expression of 1,026 genes (CKi/CK). GT represents the values from transgenic seedlings grown under normal conditions without inoculation, CK and CKi represent control seedlings without or with inoculation, respectively. **(B)** 239 differentially expressed genes (DEGs) were overlapped between CKi/CK and GT/CK. Gene Ontology (GO) **(C)** and Kyoto Encyclopedia of Genes and Genomes (KEGG) pathway **(D)** functional analyses of the DEGs from GT/CK. Photosynthesis-related processes were reduced, and defense response-related biological processes were enhanced by *ZmGT-3b* knockdown.

To investigate the biological processes and genes regulated by ZmGT-3b, RNA-seq was used to compare the transcriptomes of 7 DAG seedlings of *GT-KD* (referred to as GT) with CK. The correlation coefficient (R) for the expression profiles of all transcripts between GT and CK was 0.87, suggesting that the knockdown of *ZmGT-3b* affects overall gene transcription. We compared the transcriptional responses of *GT-KD* with CK seedlings and identified 950 DEGs (upregulated ≥ 2-fold or downregulated ≤ 2-fold; *p* <0.05), including 787 (83.7%) upregulated and 163 downregulated genes in *GT-KD* seedlings (Figure 5A). Sixteen trihelix TF genes were detected, and none of their expression levels showed a significant difference between the *GT-KD* and CK transcriptome, except *ZmGT-3b* (Supplementary Figure 4C). Of the downregulated DEG encoding proteins, 36 encode the cellular components located in photosystems, 20 in plastoglobules, 41 in photosynthetic membranes, and 37 in the plastid thylakoid. Gene Ontology (GO) and Kyoto Encyclopedia of Genes and Genomes (KEGG) analyses revealed that the downregulated DEGs were significantly enriched in photosynthesis-related functional categories, such as photosynthesis (44 DEGs), photosynthesis light reaction (30 DEGs), photosynthesis light harvesting (17 DEGs), and photosynthesis antenna proteins (16 DEGs). This suggests that ZmGT-3b is a positive regulator of photosynthesis-related processes (Figures 5C,D; Supplementary Figure 3A). Therefore, the retarded growth of *GT-KD* seedlings might be associated with the transcriptional repression of growth-promoting (photosynthesis-related) genes caused by *ZmGT-3b* knockdown.

**Figure 6:**
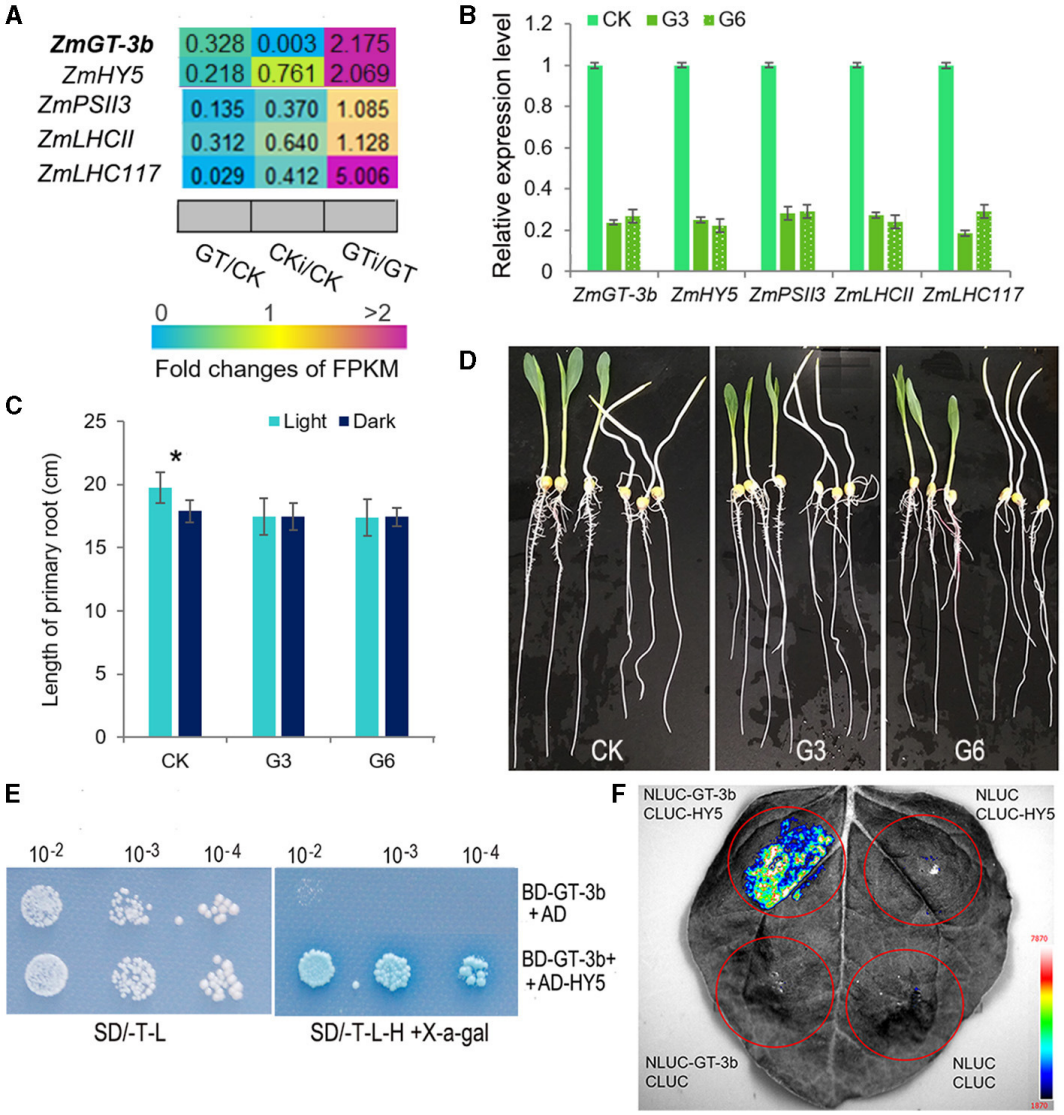
ZmGT-3b may interact with ZmHY5 to regulate photosynthesis and light-related growth. **(A)** Relative expression (fold) of photosynthesis-related genes from *ZmGT-3b* knockdown (GT) and CK (LH244) maize seedlings at 7 DAG with (CKi and GTi) or without inoculation (CK and GT) based on transcriptome sequencing. **(B)** The expression of light-responsive genes, such as *ZmGT-3b, ZmHY5, ZmPSII3, ZmLHCII*, and *ZmLHC117*, was significantly decreased in *GT-KD* seedlings, as confirmed by quantitative reverse-transcription PCR (qRT-PCR). CK represents control seedlings (LH244), G3 and G6 represent seedlings from the different transgenic events with the knocked-down expression of *ZmGT-3b*. All seedlings were grown under normal conditions and sampled at 7 DAG. **(C,D)** *GT-KD* seedlings with a significant reduction in *ZmHY5* expression caused by *ZmGT-3b* knockdown showed a disrupted shoot illumination-promoted root growth. The average primary root length of CK seedlings grown in the light was significantly higher than that of plants grown in the dark, whereas the average primary root lengths of *GT-KD* seedlings (G3 and G6) were similar in both the light and dark at 7 DAG **(C,D)**. Values are the mean ± SD (*n* > 15 individual seedlings). The asterisk * represents a significant difference at *p* < 0.05 (according to the paired Student's *t*-test). ZmGT-3b could interact with ZmHY5 in yeast **(E)** in planta **(F)**. **(E)** The AH109 yeast cells transformed with both BD-GT-3b and AD-HY5 could grow well on the medium without Trp, Leu, and His and turn the transformed yeast cell into blue, while the cells containing the BD-GT-3b and AD construct could not grow. **(F)** Luciferase complementation image (LCI) assay showed that fluorescence signal could be observed in *N. benthamiana* leaf tissues infiltrated with a combination of *Agrobacterium* cells that produce NLUC-GT-3b and CLUC-HY5. The combination of NLUC-GT-3b and CLUC, NLUC and CLUC-HY5, NLUC, and CLUC were used as negative controls.

Among the significantly downregulated genes induced by *ZmGT-3b* knockdown, the bZIP TF gene *ZmHY5* (*ELONGATED HYPOCOTYL5*) showed an identical expression profile to *ZmGT-3b*; this gene was significantly downregulated in non-inoculated *GT-KD* seedlings and upregulated in inoculated *GT-KD* seedlings (Figure 6A). Consistently, seven GT1 CONSENSUS (S000198) sites were discovered within 2,000 bp upstream of the start codon ATG of *ZmHY5*, which is the conserved DNA-binding site of GT factors (Supplementary Table 1). To verify the RNA-seq data, we performed qRT-PCR to compare gene expression levels between *GT-KD* and CK seedlings. *ZmGT-3b, ZmHY5*, and randomly selected photosynthesis-related genes, such as *ZmPSII3, ZmLHCII*, and *ZmLHC117*, were all significantly downregulated in various *GT-KD* seedlings (Figure 6B). Moreover, the primary roots of CK seedlings grown in the light were significantly longer than those grown in the dark, whereas the primary root lengths of *GT-KD* seedlings, which had a significantly reduction in the *ZmGT-3b* and *ZmHY5* expression, were similar in plants grown in the light and dark (Figures 6C,D). These suggest that the knockdown of *ZmGT-3b* led to a reduced *ZmHY5* expression, which disrupted the promotion of root growth *via* the shoot illumination in *GT-KD* seedlings. These findings are consistent with the reduced chlorophyll contents and photosynthetic rates of *GT-KD* seedlings (Figures 2D–H), suggesting that ZmGT-3b may regulate seedling growth by modulating chlorophyll biosynthesis and photosynthetic activity *via* the transcriptional regulation of photosynthesis-related genes.

HY5 lacks its own activation domain, and the observed transcriptional activation is suggested to be driven by preferential interaction with activating light- and growth-related TFs (Gangappa and Botto, 2016; Burko et al., 2020). ZmGT-3b is hypothesized to be such a TF. To investigate the interaction of ZmGT-3b and ZmHY5, we cloned the *ZmHY5* transcript that encodes 180aa, made an AD-HY5 construct, and transform it into AH109 yeast cells with BD-GT-3b simultaneously. The yeast cells containing both BD-GT-3b and AD-HY5 could grow well on the medium without His and turned the cells into a moderate blue color, whereas the cells containing BD-GT-3b and AD construct could not grow or turned the cells into blue (Figure 6E). Moreover, the interaction between ZmGT-3b and ZmHY5 was further confirmed by the luciferase complementation image (LCI) assay, a strong fluorescence signal could only be observed in *N. benthamiana* leaf tissues infiltrated with a combination of *Agrobacterium* cells that produce NLUC-GT-3b and CLUC-HY5 (Figure 6F). These indicate that ZmGT-3b could interact with ZmHY5 in yeast and in planta.

### *ZmGT-3b* Knockdown Induces Defense-Related Transcriptional Reprogramming

Most proteins encoded by the upregulated DEGs were associated with the membrane, cell periphery, or cytoplasmic vesicle, and with molecular functions, including catalytic activity, oxidoreductase activity, transferase activity, metal ion binding, cation binding, and iron ion binding. The upregulated DEGs were enriched in oxidation-reduction processes, secondary metabolic processes, plant-type cell wall organization, and reactive oxygen species (ROS) metabolic processes. These upregulated DEGs significantly contribute to the biosynthesis of secondary metabolites, especially the biosynthesis of phenylpropanoid, stilbenoid, diarylheptanoid, gingerol, benzoxazinoid, flavonoid, diterpenoid, flavone, flavonol, and carotenoid (Figures 5C,D). Almost all of these functional categories support basal defense responses to various biotic/abiotic stresses, suggesting that ZmGT-3b may act as a negative regulator of plant defense response-related biological processes.

Dramatic transcriptional reprogramming occurs during the induction of plant immune responses, allowing the plant to prioritize defense- over growth-related cellular functions. The correlation coefficient (R) for the expression profiles of all transcripts between the inoculated CK (CKi) and CK (CKi/CK) was 0.86, i.e., closer to the value of 0.87 between GT and CK (GT/CK). This indicates that inoculation and *ZmGT-3b* knockdown (GT/CK) have similar effects on general gene transcription. Compared with untreated CK seedlings, inoculation induced 1,026 DEGs, including 239 DEGs that were simultaneously induced by *ZmGT-3b* knockdown. This indicates that overlapping events or defense signaling pathways control gene expression in response to inoculation (CKi/CK) or *ZmGT-3b* knockdown (GT/CK) (Figure 5B).

The upregulated DEGs induced by inoculation (CKi/CK) were also significantly enriched in GO terms associated with the biosynthesis of secondary metabolites, especially the biosynthesis of phenylpropanoid, stilbenoid, diarylheptanoid, and gingerol, benzoxazinoid, flavonoid, diterpenoid, flavone and flavonol, carotenoid, phenylalanine metabolism, and plant-pathogen interaction. These are similar to the transcriptome reprogramming induced by *ZmGT-3b* knockdown (GT/CK), which had more DEGs enriched in photosynthesis, RNA transport, and messenger RNA (mRNA) surveillance pathway (Supplementary Figure 4). Therefore, considerable commonalities were detected in the transcriptional reprogramming induced by *ZmGT-3b* knockdown or inoculation. *ZmGT-3b* knockdown induced defense-related transcriptional reprogramming to upregulate or activate basal defense-related genes. Therefore, ZmGT-3b might act as a transcriptional repressor of genes involved in basal defense responses.

Furthermore, 1,049 genes were differentially expressed in the inoculated *GT-KD* vs. inoculated CK seedlings (GTi/CKi) (Supplementary Figure 5A). Of these, 254 DEGs were shared between the inoculated transcriptome pair GTi/CKi and the non-inoculated transcriptome pair GT/CK (Supplementary Figure 5B). The most highly enriched GO functional categories were similar, and the most highly enriched KEGG pathways were shared between the two transcriptome pairs. The transcriptional differences between the non-inoculated GT/CK transcriptome were emphasized in downregulated photosynthesis-related processes. Most of the shared functional categories are involved in basal defense responses to various biotic/abiotic stresses (Supplementary Figures 5C,D), pointing to considerable commonalities in their transcriptional responses.

However, the R for the expression profiles of all transcripts between CKi and CK was 0.86, and that between GTi and GT was 0.63, indicating that inoculation affected overall gene transcription to different degrees in the two genotypes. Compared with non-inoculated seedlings, inoculation induced the expression of 1,026 and 1,340 DEGs in CK and *GT-KD*, respectively, with 462 genes induced in both genotypes (Supplementary Figure 5B). This suggests that overlapping signaling pathways control gene expression in these two types of seedlings in response to inoculation. The transcriptional reprogramming was much stronger between inoculated and non-inoculated *GT-KD* seedlings (GTi/GT) than between inoculated and non-inoculated CK seedlings (CKi/CK). Many more DEGs were enriched in almost all top defense-related GO and KEGG functional categories, except for the biosynthesis of brassinosteroids and zeatin, which had more DEGs enriched in CKi/CK (Supplementary Figures 5E,F). These are consistent with the results that inoculated *GT-KD* have better disease resistance and lower DSI than CK seedlings.

### *ZmGT-3b* Knockdown Leads to the Upregulation of Defense-Related Genes

**Figure 7:**
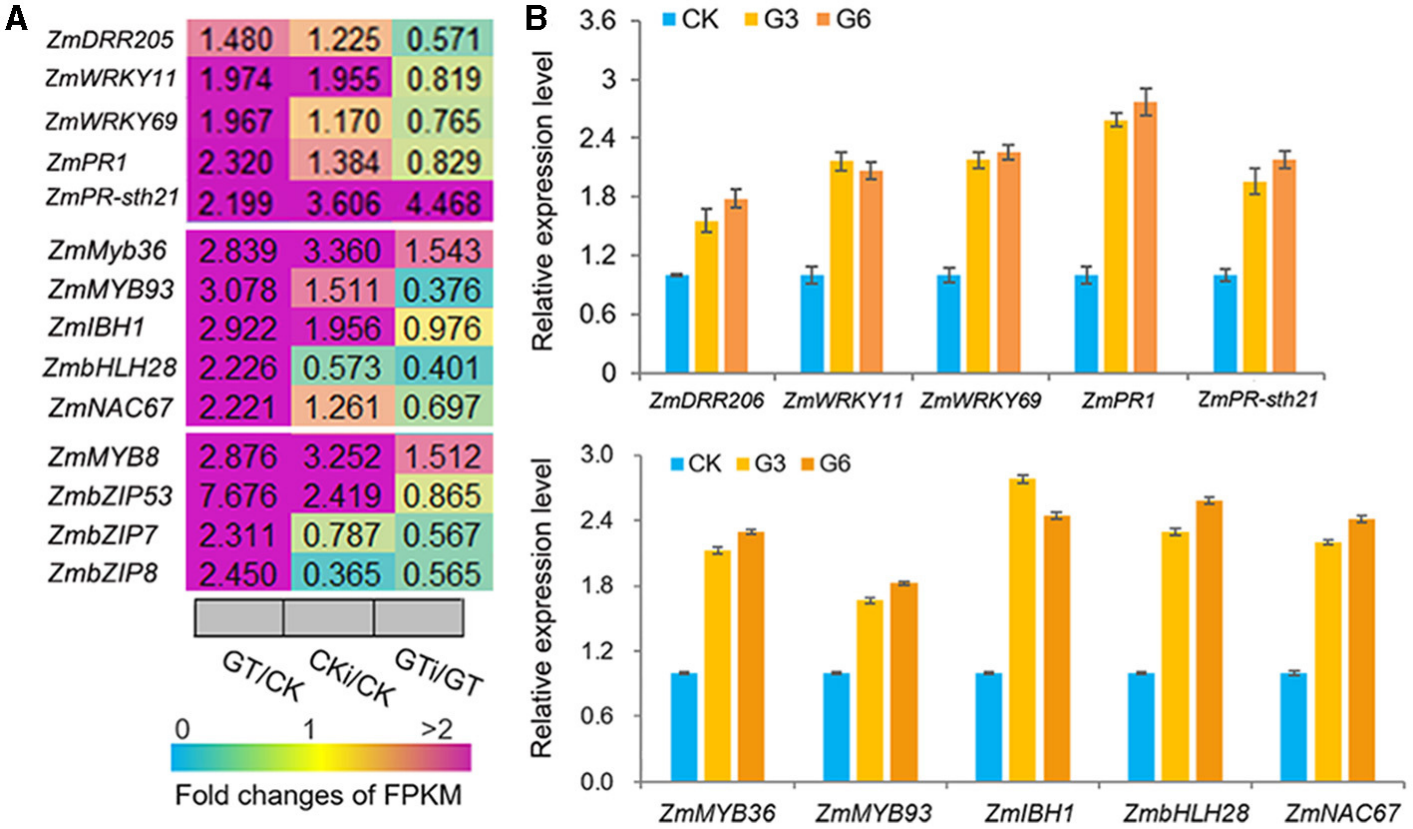
*ZmGT-3b* knockdown significantly upregulates the different transcription factor (TF) genes that are involved in the plant defense response to various biotic/abiotic stresses. **(A)** Relative expression levels (fold) of TF genes, including myeloblastosis (MYBs), basic leucine zipper (bZIPs), NAM, ATAF, and CUC (NACs), basic helix-loop-helixes (bHLHs) genes, and two *PR* genes, as revealed by the transcriptome sequencing of *ZmGT-3b* knockdown (GT) and CK (LH244, wild type) maize seedlings with (CKi and GTi) or without inoculation (CK and GT). **(B)** Defense-related genes such as *ZmDRR206, ZmPR1*, and *ZmPR-STH21* and multiple defense-related TF genes such as *ZmWRKY11, ZmWRKY69, ZmMYB36, ZmMYB93, ZmIBH1, ZmbHLH28*, and *ZmNAC67* were upregulated in *GT-KD* seedlings, as confirmed by qRT-PCR. Values are the mean ± SD (*n* = 3). CK represents CK seedlings (LH244), and G3 and G6 represent the seedlings from the different transgenic events with the knocked-down expression of *ZmGT-3b*. These experiments were repeated with three different biological repeats and each with three technical repeats. All seedlings were grown under normal conditions and sampled at 7 DAG.

*ZmGT-3b* knockdown dramatically upregulated the genes that function in defense responses to various biotic/abiotic stress, including the genes encoding 18 MYBs, eight NACs, eight ethylene-response factors (ERFs, the largest group of the AP2/EREBP family), six WRKYs, three bZIPs, three basic helix-loop-helixes (bHLHs), and six PR proteins, which were all significantly upregulated in *GT-KD* vs. CK seedlings (Supplementary Figures 3B,C). Many members of these TF families reportedly function in plant responses to various biotic or abiotic stresses. Among the TF genes upregulated by *ZmGT-3b* knockdown, some were significantly upregulated in *GT-KD* seedlings without inoculation and in CK seedlings with inoculation, contrasting to *ZmGT-3b*, including *ZmWRKY11, ZmWRKY69, ZmMYB36, ZmMYB93, ZmIBH1* (bHLH)*, ZmbHLH28, ZmNAC67, ZmMYB8, ZmbZIP53, ZmbZIP7*, and *ZmbZIP8* (Figure 7A). The upregulated expression of these TF genes and a few well-known defense-related genes such as *ZmDRR206, ZmPR1*, and *ZmPR-STH21* was confirmed by qRT-PCR in different *GT-KD* seedlings, compared with CK seedlings (Figure 7B). Therefore, various TF genes and defense-related genes were upregulated by *ZmGT-3b* knockdown.

### *ZmGT-3b* Knockdown Increases the Biosynthesis of Cell Wall Components

**Figure 8:**
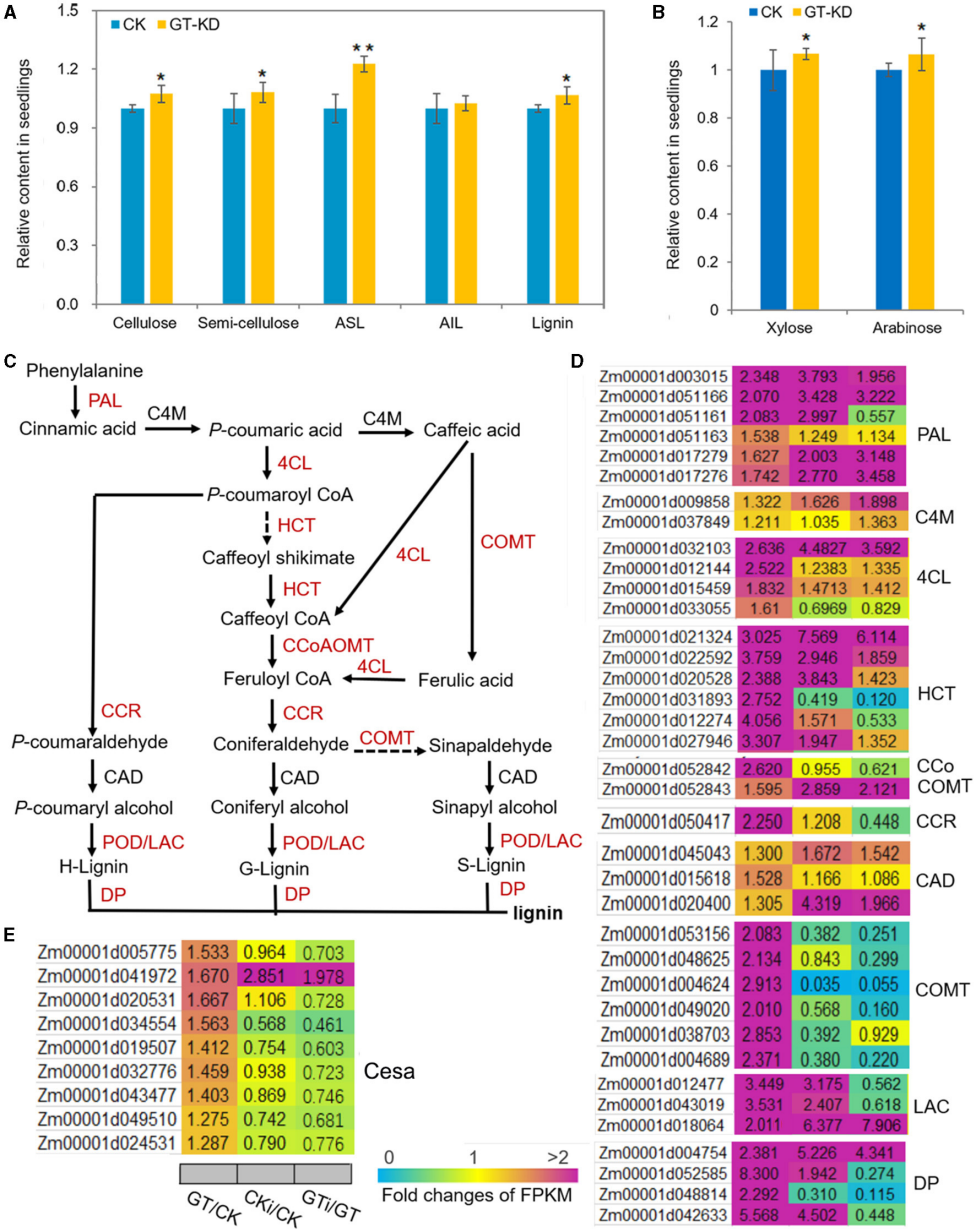
The contents of the major plant cell wall components and critical genes in the lignin biosynthesis pathway are upregulated in *ZmGT-3b* knockdown maize seedlings. **(A)** Cellulose, semi-cellulose, acid-soluble lignin (ASL), and lignin contents were significantly higher in 12 DAG *GT-KD* vs. CK (LH244, wild type) seedlings, while the acid-insoluble lignin (AIL) contents were similar. **(B)** Both arabinose and xylose levels were significantly higher in *GT-KD* seedlings than in CK seedlings. Values are the mean ± SD (*n* = 3). The asterisks represent a significant difference at *p < 0.05, **p < 0.01 (according to the paired Student's *t*-test). **(C)** The general lignin biosynthesis pathway includes the related enzymes. Red indicates significantly upregulated, and black indicates not significantly upregulated, in *GT-KD* seedlings. **(D)** Relative expression levels (fold) of genes are involved in lignin biosynthesis **(D)** and cellulose-synthase genes **(E)** in *GT-KD* seedlings. The data were obtained by transcriptome sequencing of *ZmGT-3b* knockdown (GT) and CK seedlings with (CKi and GTi) or without inoculation (CK and GT). PAL, phenylalanine ammonia-lyase; C4M, cinnamate 4-monooxygenase; 4CL, 4-coumarate CoA ligase; HCT, hydroxycinnamoyl-CoA shikimate/quinate hydroxycinnamoyl transferase; CCoAOMT, caffeoyl-CoA *O*-methyltransferase; CCR, cinnamoyl-CoA reductase; CAD, cinnamyl alcohol dehydrogenase; COMT, caffeic acid *O*-methyltransferase; LAC, laccase; POD, peroxidase; DP, dirigent protein (DP).

Based on the analysis of transcriptome reprogramming induced by *ZmGT-3b* knockdown (GT/CK), many proteins encoded by the DEGs are involved in the biosynthesis of secondary metabolites, especially phenylpropanoid (Figures 5C,D), which is the first critical step for lignin biosynthesis. Lignin is one of the most important secondary metabolites, and defense-induced lignin biosynthesis plays a major role in basal immunity. We, therefore, measured the contents of the major components of the plant cell wall, such as cellulose, semi-cellulose, and lignin in seedlings. Compared with CK seedlings, the contents of cellulose (~7.4% increase), semi-cellulose (~8.3% increase), acid soluble lignin (ASL, ~22.7% increase), and lignin (~6.64% increase) were markedly higher in *GT-KD* maize seedlings, whereas the content of acid-insoluble lignin (AIL) was not (Figure 8A). Arabinose levels are positively associated with lignin levels, and high concentrations of xylose are important in defense responses (Li F. et al., 2015). The levels of both arabinose (~6.31% increase) and xylose (~6.7% increase) were significantly higher in *GT-KD* than CK seedlings (Figure 8B). Consistently, almost all genes encoding the critical enzymes in the lignin biosynthesis pathway were significantly upregulated in *GT-KD* seedlings, including genes encoding three phenylalanine ammonia-lyases (PALs), two 4-coumarate CoA ligases (4CLs), six hydroxycinnamoyl-CoA shikimate/Quinate hydroxycinnamoyl transferases (HCTs), one caffeoyl-CoA *O*-methyltransferase (CCoAOMT), one cinnamoyl-CoA reductase (CCR), six caffeic acids *O*-methyltransferases (COMTs), three laccases (LACs), four dirigent proteins (DPs), and 23 peroxidases (PODs); genes encoding cinnamate 4-monooxygenase **(**C4M**)**, and cinnamyl alcohol dehydrogenase (CAD) were not upregulated in these seedlings. Seven genes encoding Casparian strip membrane proteins (CASPs) were also significantly upregulated in *GT-KD* vs. CK seedlings (Figures 8C,D; Supplementary Figure 3). Furthermore, all 22 cellulose-synthase (Cesa) genes with highly abundant transcripts (with FPKM > 20) showed elevated expression levels in *GT-KD* seedlings, and 4 of these genes (with FPKM > 50) had FC > 1.5 in *GT-KD* vs. CK seedlings (Figure 8E). These findings suggest that *ZmGT-3b* knockdown promoted secondary metabolite biosynthesis, especially lignin biosynthesis, which occurred in maize seedlings grown under the normal condition without fungus infection.

Many genes upregulated in *ZmGT-3b* knockdown plants encode proteins involved in metal ion binding, cation binding, or iron ion binding (Figures 5C,D). Thus, we measured the contents of mineral elements in CK and *GT-KD* seedlings. Compared with CK seedlings, potassium (K^+^), phosphorus (P), and copper (Cu) levels were significantly higher, whereas aluminum (Al) and iron (Fe) levels were significantly lower, in *GT-KD* seedlings. However, Zn, Mg, and Na levels did not significantly differ in *GT-KD* vs. CK seedlings (Supplementary Figure 6A). Consistent with the different contents of various mineral elements, genes encoding transporters of these elements showed considerably different transcript levels in *GT-KD* vs. CK seedlings, including genes encoding phosphate, K^+^, Cu, vacuolar Fe, and zinc transporters. Two sulfate transporter genes were significantly upregulated in *GT-KD* vs. CK seedlings (Supplementary Figure 6B). These findings suggest that *ZmGT-3b* knockdown altered cellular osmotic conditions by affecting the take-up of inorganic ions from the environment using the corresponding transporters.

## Discussion

The growth–defense trade-off in plants is associated with the limited availability of resources, which requires the plant to prioritize growth or defense, depending on dynamic external and internal factors. This balance is important for agriculture and natural ecosystems due to the vital importance of these processes for plant survival, reproduction, and plant fitness (Huot et al., 2014). Numerous studies have revealed the crucial roles of TFs in regulating plant growth, development, and biotic or abiotic stress responses, including the trihelix TFs (Kaplan-Levy et al., 2012). *OsGT*γ*-2* overexpressing could improve the seed germination rate, seedling growth, and SR of the transgenic plants under salinity stress (Liu et al., 2020). *ZmGT-3b* knockdown suppressed seedling growth, reduced photosynthesis activity, and downregulated the photosynthesis-related genes, with synchronically activated constitutive defense responses, increased disease resistance to *F. graminearum*, and significantly upregulated many defense-related genes in maize seedlings; thus, these finally caused a growth–defense trade-off phenotype of the *GT-KD* seedlings (Figures 2–7; Supplementary Figure 3). These findings suggest that ZmGT-3b might involve in pathogen attack-induced suppression of photosynthesis activity, and coordinates metabolism during growth–defense trade-off by optimizing the temporal and spatial expression of photosynthesis- and defense-related genes, uncovering a molecular mechanism underlying the growth–defense trade-off.

### ZmGT-3b Modulates Seedling Growth *via* the Transcriptional Regulation of Photosynthesis-Related Genes

The conserved DNA-binding domains of plant-specific trihelix TFs are often found at the N-terminus. By contrast, the C-terminus, which harbors a hydrophilic region, is less conserved and probably acts as the activation domain (Kaplan-Levy et al., 2014; Qin et al., 2014). Trihelix TFs control the transcription of light-regulated genes as well as the genes involved in plant development and stress responses (Kaplan-Levy et al., 2012). During these transcriptional regulatory processes, trihelix TFs form homodimers or heterodimers by participating in interactions with other classes of TFs (Xie et al., 2009; Li B. et al., 2015). Contrast to the light-independent expression of *AtGT-1* (Qin et al., 2014), the expression of *ZmGT-3b* was light-inducible, consistent with the identified 19 light-responsive elements in the promoter of this gene (Figure 1A; Supplementary Table 1).

Due to the deficiency of correctly annotated information of *ZmGT-3b* in 2013, we obtained *GT-KD* mutants with severely reduced *ZmGT-3b* transcript levels by transforming the maize inbred line LH244 with a cDNA fragment encoding the C-terminal 149 aa of ZmGT-3b under the control of maize *Ubiquitin* promoter, although we had planned to obtain *ZmGT-3b* overexpression maize plants at the beginning. The reason for the *ZmGT-3b* knockdown resulted from the transformed partial cDNA of *ZmGT-3b* remains elusive although we could provide evidence for a significantly reduced *ZmGT-3b* transcript levels (the reduction of both the endogenous and the transformed transcripts) by multiple times qRT-PCR experiments with different pairs of specific primers and transcriptome data. That is, the overall transcript of *ZmGT-3b* (including the endogenous and the transformed partial cDNA) was significantly reduced in *GT-KD* seedlings (Figure 2). There might be a co-suppression mechanism that occurred in *GT-KD* seedlings as various public transcriptome data revealed that *ZmGT-3b* only expressed in few kinds of young tissues with low levels. The strong maize *Ubiquitin* promoter might induce abundant partial transcripts of *ZmGT-3b* that finally led to co-suppression and a decreased endogenous *ZmGT-3b* level. Similarly, the *SlGA20ox1* gene was silenced by its 622 bp coding sequence, which was ligated in sense orientation downstream to the *CaMV 35S* promoter for generating the co-suppression vector, and *GA20ox1* was co-suppressed in the obtained transgenic tomato plants that showed vegetative defects typical of GA deficiency such as darker and misshaped leaves and dwarfism (Olimpieri et al., 2011). *Arabidopsis* plants were originally engineered to overexpress *MIPS2*, but ultimately in fact reduced the expression of *AtMIPS1, AtMIPS2*, and *AtMIPS3*, the co-suppression of their expression levels induced the transgenic plants with altered vegetative phenotypes, reduced inositol, and increased glucose levels (Fleet et al., 2018).

Light perception activates many TFs from various families, such as bZIP, bHLH, MYB, GATA, and GT1. These TFs bind to various LREs, such as G, GT1, GATA, and MREs, leading to massive transcriptional reprogramming (Gangappa and Botto, 2014, 2016). Among these TFs, the role of HY5 as a master transcriptional regulator is conserved across plant species. HY5 mediates the light-responsive coupling of shoot growth and photosynthesis with root growth and nitrate uptake, and it functions as the center of a transcriptional network hub connecting different processes such as hormone, nutrient, abiotic stress (ABA, salt, cold), and ROS signaling pathways (Chen et al., 2016; Gangappa and Botto, 2016; Burman et al., 2018). HY5 activates its own expression and is a critical player in seedling development and responses to light by turning on or off many genes involved in fundamental developmental processes such as cell elongation, pigment accumulation (chlorophyll and anthocyanin), flowering, and root development (Kobayashi et al., 2012; Toledo-Ortiz et al., 2014; Chen et al., 2016). The activator or repressor activity of HY5 in the transcription regulation during plant growth and light responses depends on its interacting partners as HY5 does not have its own activation or repression domain. The primary function of HY5 in promoting transcription may depend on other, likely light-regulated, factors (Burko et al., 2020). *ZmGT-3b* knockdown induced the downregulated DEGs to be significantly enriched in the functional category photosystem, but not in carbon metabolism (Figures 5C,D; Supplementary Figure 3). The significantly downregulated expression of *ZmHY5*, the reduced Pn, and the disruption of the effect of shoot illumination on promoting the root growth of the *GT-KD* seedlings, and the interaction of ZmGT-3b and ZmHY5 in yeast and in planta (Figure 6), suggesting that ZmGT-3b may function as a novel interacting light-/growth-related partner for ZmHY5, to coordinately regulate the transcription of photosynthesis-related genes during the young seedling growth.

### *ZmGT-3b* Knockdown Induces Constitutive Defense Responses by Regulating Defense- and Lignin Biosynthesis-Related Gene Expression

Diminished growth is thought to be an integral facet of induced resistance and a molecular mechanism involved in the cross talk between growth and defense responses. This process involves the optimization of the temporal and spatial expression of defense genes. Pathogen infection usually affects primary metabolism, reduces plant growth, limits photosynthesis, and modifies secondary metabolism toward defense responses (Guo et al., 2018). ASR3 is reported to be a negative regulator of PTI (Li B. et al., 2015), whereas GTL1 is a positive regulator of defense genes and negatively associated with plant growth and development (Völz et al., 2018). *ZmGT-3b* was positively associated with seedling growth and photosynthesis, whereas it was negatively associated with disease resistance, drought tolerance, and cell wall component biosynthesis (Figures 2–4, 8). Moreover, the transcriptome reprogramming induced by *ZmGT-3b* knockdown was similar to that induced by *F. graminearum* fungus infection in CK seedlings (CKi/CK) (Supplementary Figure 4). Many upregulated genes encoded multiple members of the MYB, bZIP, WRKY, NAC, ERF, and bHLHs TF families (Supplementary Figure 3C). Some members of these TF families are the well-known regulators of lignin biosynthesis, particularly MYBs are the important regulators of both secondary cell wall biosynthesis and abiotic stress tolerance (Mizoi et al., 2012; Baldoni et al., 2015). NAC-MYB-GRN regulates lignin biosynthesis in both dicot and monocot species (Yoon et al., 2015). Five maize MYB TFs (ZmMYB2, ZmMYB8, ZmMYB31, ZmMYB39, and ZmMYB42) function in lignin biosynthesis by controlling *ZmCOMT* expression (Fornale et al., 2006). The overexpression of *ZmMYB167* increased the lignin content to 13% in maize without affecting plant growth or development (Bhatia et al., 2019). Besides MYB TFs, some NAC (Zhong et al., 2007) and WRKY TFs also regulate lignin biosynthesis by modulating the expression of cell wall synthesis-related genes (Gallego-Giraldo et al., 2016).

The plant cell wall is a highly organized and dynamic structure and composed of lignin, cellulose, semi-cellulose, pectin, proteins, and aromatic substances, it functions not only as a passive defensive barrier but also as an essential component of plant monitoring systems. Cell wall biosynthesis requires the coordinated action of numerous enzymes that are often coordinately regulated both spatially and temporally by specific TFs (Liu et al., 2018; Ohtani and Demura, 2019). Increasing lignin contents *via* the activation of the immune response is a conserved basal defense mechanism in plants, allowing defense-induced lignification to be used as a biochemical marker of an activated immune response (Dixon and Barros, 2019; Vanholme et al., 2019). In addition to well-known enzymes involved in monolignol biosynthesis, DPs, PODs, and LACs are the components of the lignin polymerization machinery (Liu et al., 2018). Many genes encoding these enzymes are induced by various biotic and abiotic stresses, highlighting their roles in the biosynthesis of defensive lignin and/or strengthening of the cell wall *via* lignin deposition in response to stress (Paniagua et al., 2017). *ZmGT-3b* knockdown led to a significant upregulation of a subset of secondary metabolite biosynthesis-related genes, especially genes encoding lignin biosynthesis enzymes, including PALs, 4CLs, HCTs, CoCOMTs, CCR, COMTs, LACs, DPs, PODs, and CASPs, in *GT-KD* vs. CK seedlings (Figure 8D; Supplementary Figure 3B). *ZmMYB19/Zm1* is an ortholog of the *Arabidopsis* SG3-type R2R3-MYB genes *MYB58* and *MYB63*. These TFs transactivate the promoters of monolignol pathway genes, and their overexpression specifically activates the monolignol pathway and lignin accumulation at the expense of biomass production (Zhou et al., 2009). Among the 18 MYB genes with significantly upregulated levels in *GT-KD* vs. CK seedlings, *ZmMYB19/Zm1* expression increased 2.04-fold in response to *ZmGT-3b* knockdown (Supplementary Figure 3C). The upregulation of these genes might contribute to the increased ASL and lignin contents of *GT-KD* seedlings (Figure 8A). Lignin biosynthesis is coordinately regulated with the biosynthesis of other cell wall components. The deposition of cellulose in cell walls is vital for controlling cell growth (Yoon et al., 2015; Liu et al., 2018; Ohtani and Demura, 2019). BdTHX1 is reported to involve in the regulation of MLG biosynthesis by controlling the transcription of *BdCSLF6* (Fan et al., 2018). Consistently, the contents of cellulose and semi-cellulose were also higher in *GT-KD* seedlings compared with CK seedlings (Figure 8A). These suggest that *ZmGT-3b* knockdown induces constitutive defense responses without fungus infection by transcriptionally regulating basal defense- and lignin biosynthesis-related genes in maize seedlings.

### Enhanced Drought Tolerance by *ZmGT-3b* Knockdown in Maize Seedlings

Drought severely limits crop productivity worldwide. Drought causes plant dehydration by disrupting cellular osmotic equilibrium, ultimately leading to various physiological and metabolic disorders such as damaged photosynthetic activity and excess ROS production. Plants have evolved various mechanisms to protect themselves from drought stress or to tolerate dehydration. Such mechanisms include the biosynthesis of various low molecular weight osmotic-protective compounds, maintaining cell water content with ion accumulation, reinforcing the cell wall, and detoxifying ROS (Mak et al., 2014; Tenhaken, 2015; Zhao et al., 2018). The biosynthesis and accumulation of osmotic-protective compounds is an energy-consuming process. Increasing cytosolic ion concentrations by taking up inorganic ions from the environment *via* transporters and channels is a much more cost-effective method for an intracellular osmotic adjustment in plants (Conde et al., 2011).

Some ion transporters are involved in the regulation of ion homeostasis and balancing ROS production. K^+^ is the most abundant cation and an essential element for plants. K^+^ is critical for the adaptive responses of plants to various abiotic or biotic stresses, including drought stress as increased K^+^ uptake confers higher levels of drought tolerance (Feng et al., 2016; Cai et al., 2019). P is an indispensable nutrient for plant growth and development as it is a constituent of many important molecules such as nucleic acids, phospholipids, and ATP (Guo et al., 2015). Cu is critical for electron transport and for scavenging ROS produced in chloroplasts during photosynthesis under stress conditions (Boutigny et al., 2014). *OsGT*γ*-2* regulates rice responses to salt stress by regulating the expression of ion transporters (Liu et al., 2020). Similarly, *ZmGT-3b* knockdown could significantly increase the contents of K, P, and Cu, and upregulated their corresponding transporter genes in *GT-KD* seedlings (Supplementary Figure 6). Sulfate transporters are important for plant drought and salinity tolerance as sulfate accumulation in leaves enhances ABA biosynthesis, leading to stomatal closure (Gallardo et al., 2014). Two sulfate transporter genes were also significantly upregulated in *GT-KD* seedlings. These might contribute to the enhanced drought tolerance of *GT-KD* seedlings. Fe is involved in various chelation and oxidation/reduction steps that affect ROS production because it is a component of all photosystems and a critical redox-active metal ion in a photosynthetic electron flow. Therefore, Fe homeostasis must be fine-tuned in plants (Briat et al., 2007). Al is toxic to plants and seriously affects plant growth and productivity (Yin et al., 2010). Finally, since lignin is a component of the cell wall and the first barrier for metal ions, lignin biosynthesis is associated with heavy metal absorption, transport, and tolerance in plants. Lignin binds to multiple heavy metal ions and reduces their entry into the cytoplasm due to its numerous functional groups (e.g., hydroxyl, carboxyl, and methoxyl) (Dalcorso et al., 2010). A significantly reduction in Al and Fe contents in *GT-KD* vs. CK seedlings might be associated with increased lignin biosynthesis in response to *ZmGT-3b* knockdown.

Members of the AP2/ERF, MYB, bZIP, and NAC TF families are involved in the transcriptional regulation of genes required for drought tolerance. MYBs are the important regulators of both secondary cell wall biosynthesis and abiotic stress tolerance, perhaps linking the abiotic stress response and lignin biosynthesis pathways. Drought induces the expression of many *MYB* genes: 65% of *MYB* genes in rice that is expressed in seedlings are differentially regulated under drought stress (Katiyar et al., 2012), and different MYB TFs are involved in one or more drought response mechanisms (Baldoni et al., 2015). *Betula platyphylla* plants overexpressing *BplMYB46* showed increased salt and osmotic stress tolerance and enhanced lignin deposition (Guo et al., 2017). Lignin helps maintain osmotic balance in the cell and protects membrane integrity by reducing the penetration of water into the plant cell and hampering transpiration, and lignin biosynthesis increases under drought stress (Mourasobczak et al., 2011). MYB15 plays a role in the complex regulatory relationship between lignin, growth, and defense (Chezem et al., 2017). Many trihelix family members, including *GmGT-2A* and *GmGT-2B, AtGTL1*, and *ShCIGT*, are reported to involve in the regulation of drought tolerance (Xie et al., 2009; Yoo et al., 2010; Yu et al., 2018). Consistent with our observation of a reduced TR and increased lignin contents in *GT-KD* seedlings (Figures 4E, 8A), *ZmGT-3b* knockdown induced a significant upregulation of multiple members of the bZIP, MYB, WRKY, NAC, ERF, and bHLH families (Supplementary Figure 3C). Considering that these TFs are critical in the complex regulatory relationship between growth, lignin, and defense, their elevated expression might be associated with the improved disease resistance and drought tolerance of *GT-KD* seedlings. Collectively, the reinforced cell wall with increased lignin content, the increased accumulation of various mineral elements, and a significant upregulation in the expression of various defense-related TFs all might contribute to the drought tolerance of *GT-KD* seedlings.

Based on these results, we propose a model for the mode of action of ZmGT-3b (shown in Supplementary Figure 7): under normal growth conditions, light induces the expression of *ZmGT-3b*. ZmGT-3b may act as a novel interacting partner of ZmHY5 to activate various photosynthesis-related genes, thereby promoting photosynthesis and seedling growth; ZmGT-3b may synchronically act as a transcriptional repressor of the expression of multiple TF genes, including *MYB*s, *bZIP*s, *NAC*s, *bHLH*s, and *ERF*s, which in turn repress the expression of various defense-related genes. When plants are exposed to a pathogen attack, the expression of *ZmGT-3b* and *ZmHY5* was dramatically decreased, thus relieving the repressive effects of these TF genes and enhancing the expression of defense-related genes, including genes encoding PR proteins and various enzymes involved in the biosynthesis of secondary metabolites, especially lignin, thereby activating the defense response. Reduced *ZmGT-3b* expression also leads to decreased photosynthesis activity to benefit defense-related biological processes. Therefore, we propose that ZmGT-3b might serve as a regulator in the coordination of the metabolism during a growth–defense trade-off by optimizing the temporal and spatial expression of photosynthesis- and defense-related genes, especially the secondary metabolite biosynthesis.

## Data Availability Statement

The datasets presented in this study can be found in online repositories. The name of the repository and accession number can be found at: National Center for Biotechnology Information BioProject, PRJNA761591 (https://www.ncbi.nlm.nih.gov/Traces/study/?acc=PRJNA761591).

## Author Contributions

JY: conceived and designed the project. QZ, JY, TZ, and LE: performed the experiments. MX: supervised the project and experiments. JY: designed the experiments, analyzed the data, and wrote the article with contributions of all the authors. All authors approved the final version of the article.

## Funding

This work was supported by the Ministry of Agriculture and Rural Affairs of the People's Republic of China (Grant No. 2018ZX0800917B) and the National Natural Science Foundation of China (31671704).

## Conflict of Interest

The authors declare that the research was conducted in the absence of any commercial or financial relationships that could be construed as a potential conflict of interest.

## Publisher's Note

All claims expressed in this article are solely those of the authors and do not necessarily represent those of their affiliated organizations, or those of the publisher, the editors and the reviewers. Any product that may be evaluated in this article, or claim that may be made by its manufacturer, is not guaranteed or endorsed by the publisher.
