# Supplementary material for: GT Factor ZmGT-3b Is Associated With Regulation of Photosynthesis and Defense Response to Fusarium graminearum Infection in Maize Seedling
Source: Front Plant Sci. 2021 Nov 18;12:724133. doi: 10.3389/fpls.2021.724133 (PMC8638620; doi:10.3389/fpls.2021.724133)
Supplement: Supplementary file 1 [file Data_Sheet_1.pdf]

SUPPLEMENTARY MATERIALs

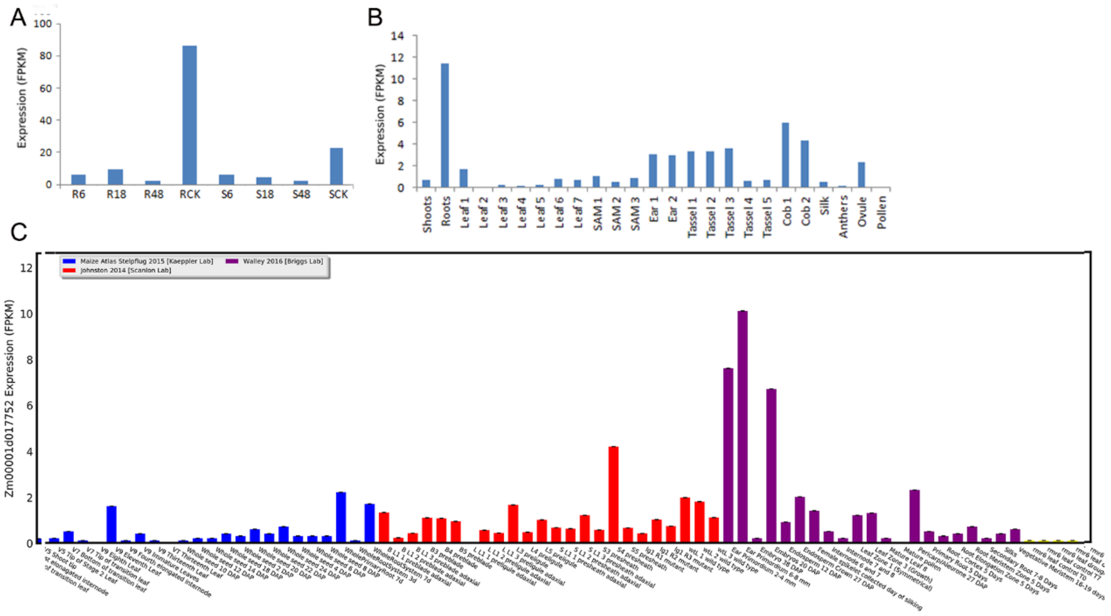

FIGURE S1 I Expression profile of *ZmGT-3b*. (A) *ZmGT-3b* expression was rapidly and dramatically decreased after inoculation in both the resistant *qRfg1* allele (R-NIL) or the susceptible *qRfg1* allele (S-NIL), which were developed from a QTL *qRfg1* on chromosome 10 that could explain 36.6% of the total variations of maize resistance to *Fusarium graminearum* induced stalk rot (Yang et al., 2010). RCK (SCK) is the primary roots of R-NIL (S-NIL) without inoculation, R6, R18, R48 (S6, S18, S48) was the primary roots of R-NIL (S-NIL) inoculated after 6h,18h and 48h, respectively (Ye et al., 2013). (B and C) *ZmGT-3b* expressed highly in normal seedling roots, and only expressed in a few kinds of young tissues, such as primary roots, ear primordium (2-8μm), embryo at 20 DAP and presheath et al (Chen et al, 2014; Johnston 2014; Maize atlas Stelpflug 2015; Walley 2016).

Supplemental literatures

Chen J, Zeng B, Zhang M, Xie S, Wang G, Hauck A, Lai J (2014) Dynamic transcriptome Landscape of Maize Embryo and Endosperm Development. Plant physio, 166, 972: 252-264

Johnston R, Wang M, Sun Q, Sylvester AW, Hake S, Scanlon MJ. Transcriptomic analyses indicate that maize ligule development recapitulates gene expression patterns that occur during lateral organ initiation. Plant Cell. 2014, 26(12):4718-32.

Stelpflug SC, Sekhon RS, Vaillancourt B, Hirsch CN, Buell CR, de Leon N, Kaeppler SM. An Expanded Maize Gene Expression Atlas based on RNA Sequencing and its Use to Explore Root Development. Plant Genome. 2016, 9(1).

Walley JW, Sartor RC, Shen Z, Schmitz RJ, Wu KJ, Urich MA, Nery JR, Smith LG, Schnable JC, Ecker JR, Briggs SP. Integration of omic networks in a developmental atlas of maize. Science. 2016, 353(6301): 814-8.

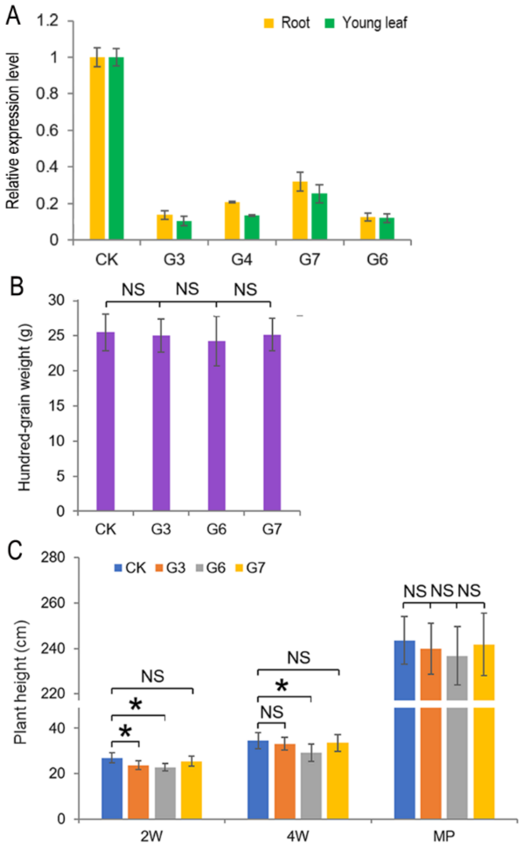

FIGURE S2 I The decreased *ZmGT-3b* transcript levels, and the maize mature kernel weight and plant height analysis. (A) The transcript levels of *ZmGT-3b* were dramatically decreased in both the roots and leaves of the *GT-KD* seedlings, compared with CK seedlings, with a pair of primers from the third exon (*ZmGT-3b*-qF2：5'- GGCAGTCGATGCAGAGGATA-3'；*ZmGT-3b*-qR2：5'-CCTGGTGAGCAAGGTAGTGAG-3') to detect transcript level of the transformed partial cDNA of *ZmGT-3b*. Values are the mean ± SD ($n = 3$). (B) The one-hundred-kernel-weight (HKW) of the *GT-KD* plants was similar to that of the CK plants. The mature kernels were harvested and sun-dried under identical conditions. Values are the mean ± SD ($n = 3$). (C) The mature *GT-KD* and CK plants grown in the same field had similar plant height. Values are the mean ± SD ($n = 3$). CK is the wild type plants (LH244)，G3，G6 and G7 were different transgenic maize plants with *ZmGT-3b* knock-down，all the plants were grew in the field under normal condition in Beijing, 2018. 2w/4w was 2/4 weeks and MP was mature plant. Three independent repeats were done in the field. Values are the mean ± SD (n=3). The asterisk * represents a significant difference at $P < 0.05$ (according to a paired Student's t-test);

NS, not significant.

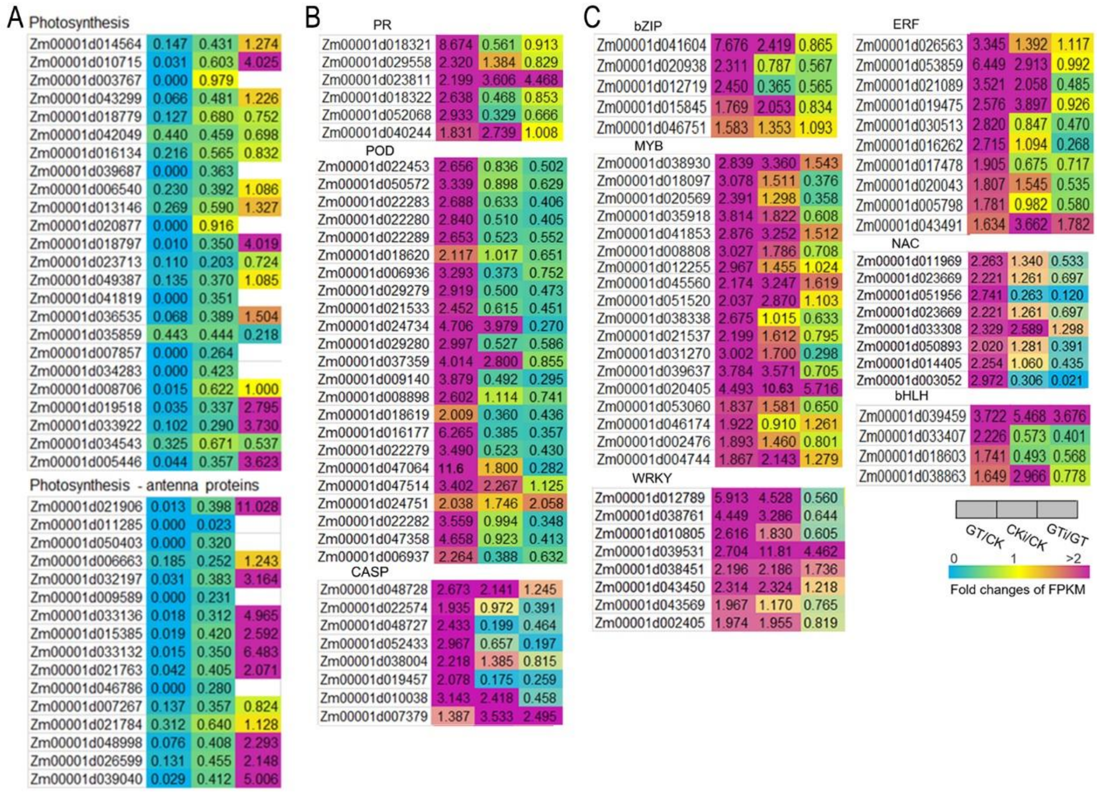

FIGURE S3 I The differential expression of the photosynthesis, defense-related and TF encoding genes in *GT-KD* seedlings. The relative expression fold of the photosynthesis-related genes (A), defense-related genes (B) and the TF encoding genes (C). The data was from transcriptome sequencing with *ZmGT-3b* knock-down (GT) and CK (LH244) maize seedlings, with (CKi, GTi) or without inoculation (CK, GT). *ZmGT-3b* knock-down significantly down-regulated genes associated with photosynthesis-related functional categories, while significantly upregulated genes that emphasized in functions in plant defense response to various biotic/abiotic stress, such as POD, PR, CASP and a few TF family member encoding genes, such as bHLH, bZIP, MYB, WRKY, ERF, and NAC.

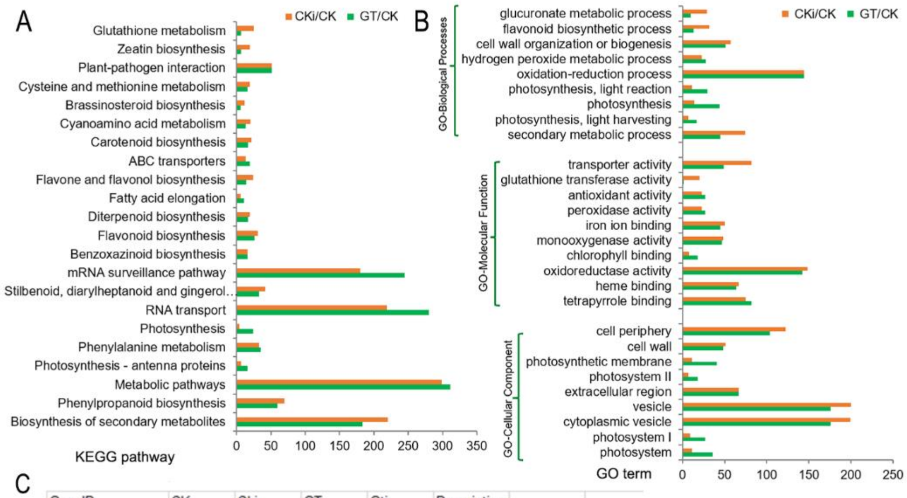

C

GeneID	CK	Cki	GT	Gti	Description
Zm00001d016876	10.701	11.653	10.993	8.813	Trihelix transcription factor ASIL2
Zm00001d035457	1.559	1.152	2.565	2.205	Trihelix transcription factor GT-3b
Zm00001d025720	3.730	4.055	5.080	3.581	Trihelix transcription factor GT-1
Zm00001d013849	6.473	5.018	8.049	5.703	Trihelix transcription factor GT-2
Zm00001d050583	1.794	1.570	3.204	2.206	Trihelix transcription factor ASR3
Zm00001d054080	4.575	6.880	3.579	5.614	Trihelix transcription factor GTL2
Zm00001d026245	2.037	2.170	3.026	1.855	Trihelix transcription factor GT-3b
Zm00001d003549	3.650	3.194	3.277	3.596	Trihelix transcription factor ASIL2
Zm00001d003889	7.631	5.417	7.403	4.287	Trihelix transcription factor ASR3
Zm00001d016604	5.097	7.242	7.901	3.788	Trihelix transcription factor ASR3
Zm00001d025514	9.173	13.761	9.018	9.897	Trihelix transcription factor ASIL2
Zm00001d032453	14.652	9.047	19.788	14.130	Trihelix transcription factor GT-2
Zm00001d014938	1.764	1.804	1.416	1.443	Trihelix transcription factor GTL2
Zm00001d053178	3.969	2.910	3.701	2.912	Trihelix transcription factor
Zm00001d027335	6.532	2.201	9.677	6.324	Trihelix transcription factor GT-2
**Zm00001d017752**	**441.471**	**1.325**	**144.977**	**315.311**	**Transcription factor GT-3b**

FIGURE S4 I Significant commonalities exist in the transcriptional responses between the *ZmGT-3b* knockdown (GT) and inoculated control plants (CKi). *ZmGT-3b* knockdown (GT) induced similar transcriptional reprogramming to the inoculated control plants (CKi), to enhance defense related functional categories and to up-regulate expression of defense-related genes by GO (A) and KEGG (B) analysis with the obtained DEGs. (C)The expression levels of other trihelix TF family members did not show significant changes in the *GT-KD* seedlings.

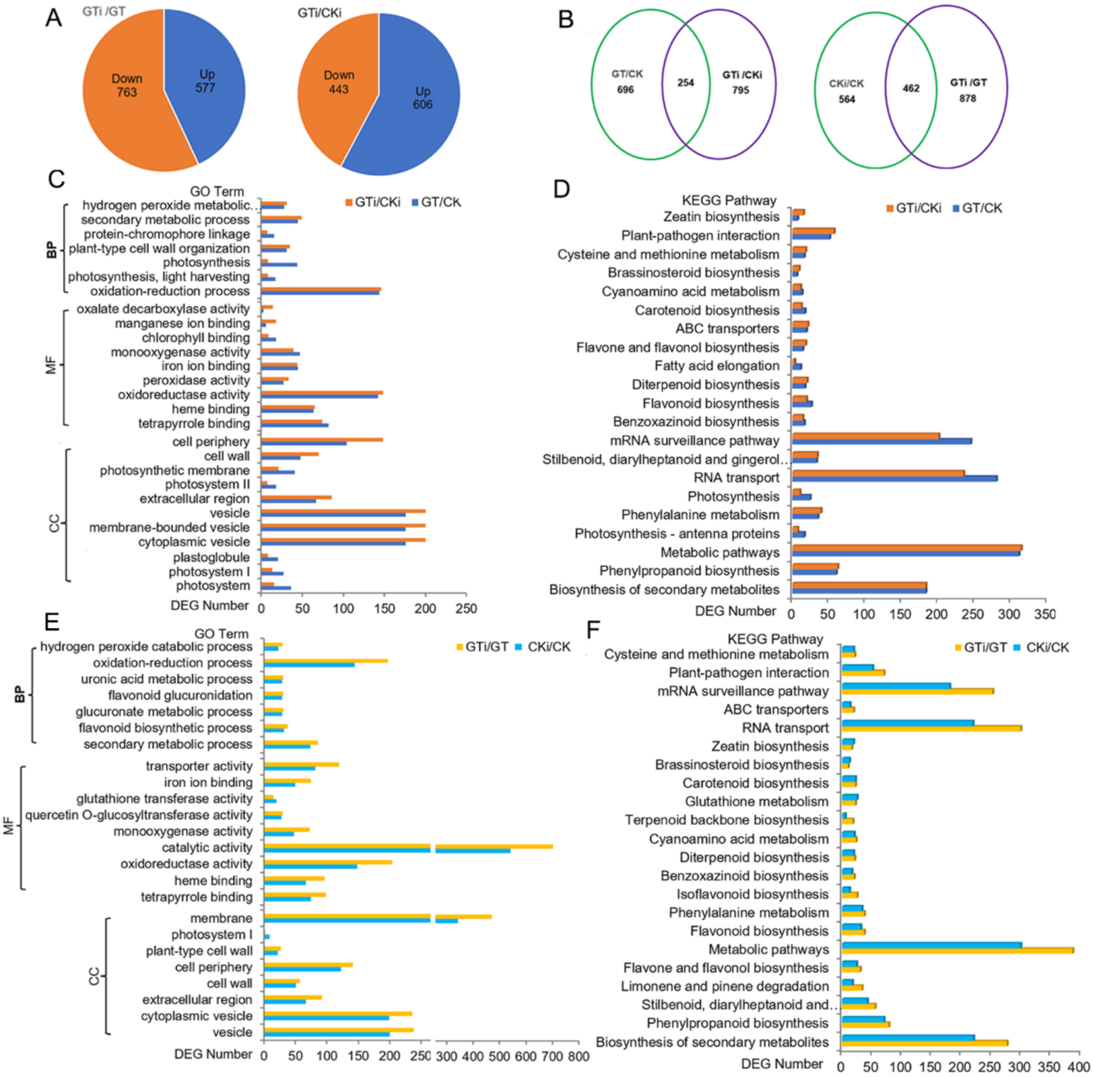

FIGURE S5 I Comparative transcriptome composition between the various transcriptome pairs. (A) Inoculation induced 1340 genes to be differently expressed in *GT-KD* seedlings (GTi/GT), while 1049 genes were differently expressed in the inoculated two genotypes (GTi/CKi). (B) 254 differentially expressed genes (DEGs) were shared between GT/CK and GTi/CKi, while 462 DEGs were shared between CKi/CK and GTi/GT. Significant commonalities exist in the transcriptional responses between the non-inoculated GT/CK and the inoculated GTi/CKi by GO (C) and KEGG (D) analysis. (E, F) The transcriptional reprogramming between the inoculated and non-inoculated *GT-KD* seedlings (GTi/GT) was much stronger than that of the inoculated and non-inoculated CK seedlings (CKi/CK).

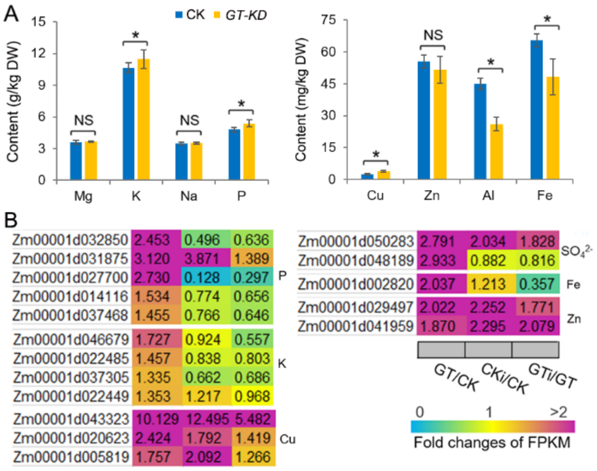

FIGURE S6 I Contents of the mineral elements and expression fold of the related transporter encoding genes in maize seedlings. (A) The contents of mineral elements in CK and *GT-KD* maize seedlings at 7-DAG. Compared with CK seedlings, the content of Al and Fe was significantly decreased, while the content of Cu, K and P was increased in the *GT-KD* seedlings. DW, Dry weight. Values are the mean ± SD (n=3). The asterisk * represents a significant difference at $P < 0.05$ (according to a paired Student's t-test); NS, not significant. (B) The relative expression fold of the related mineral element transporter encoding genes from the transcriptome sequencing with *ZmGT-3b* knock-down (GT) and CK (LH244) maize seedlings, with (CKi, GTi) or without inoculation (CK, GT).

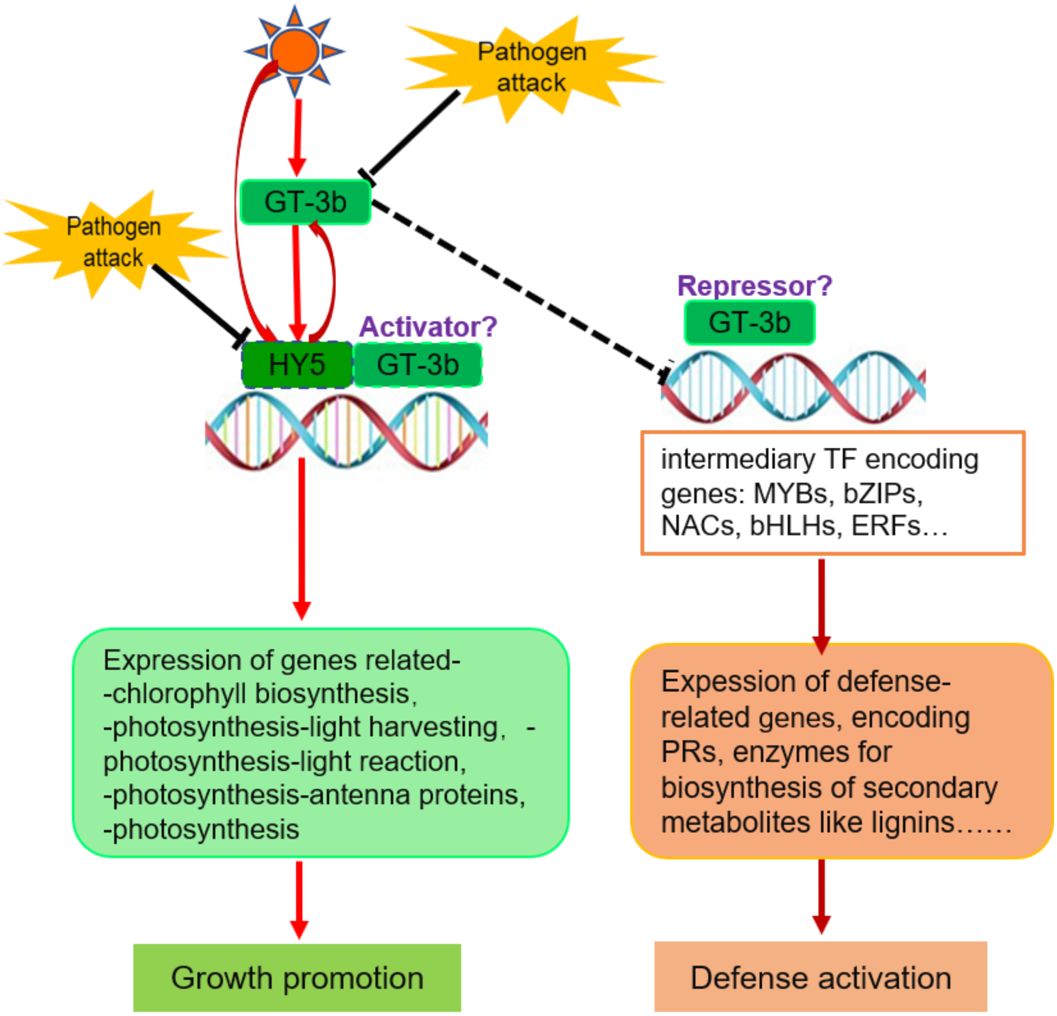

FIGURE S7 I A proposed model for the involvement of ZmGT-3b in regulating growth and defense response in maize seedlings. Under normal growth conditions, light induces *ZmGT-3b* expression, and ZmGT-3b transcriptionally activates various photosynthesis-related genes, especially *ZmHY5*, to promote photosynthesis and seedling growth; ZmGT-3b synchronically suppresses the expression of various defense-related genes by transcriptionally repressing multiple transcription factor (TF) genes, including *MYB*s, *bZIP*s, *NAC*s, *bHLH*s, and *ERF*s. However, *ZmGT-3b* and *ZmHY5* expression dramatically decreases upon pathogen attack. Thus, photosynthetic activity is limited and the repressive effect on TF genes is relieved, thereby inducing the expression of defense-related genes to activate the defense response. Thick lines indicate direct inhibition by pathogens, while broken lines indicate proposed repression. ZmGT-3b and ZmHY5 function collaboratively in regulating the light response and photosynthesis during seedling growth.

Table S1 I The cis-element analysis within 2000bp upstream of *ZmGT-3b*, *ZmHY5* starting codon ATG.

Table S2 I All the primers used in the experiments.

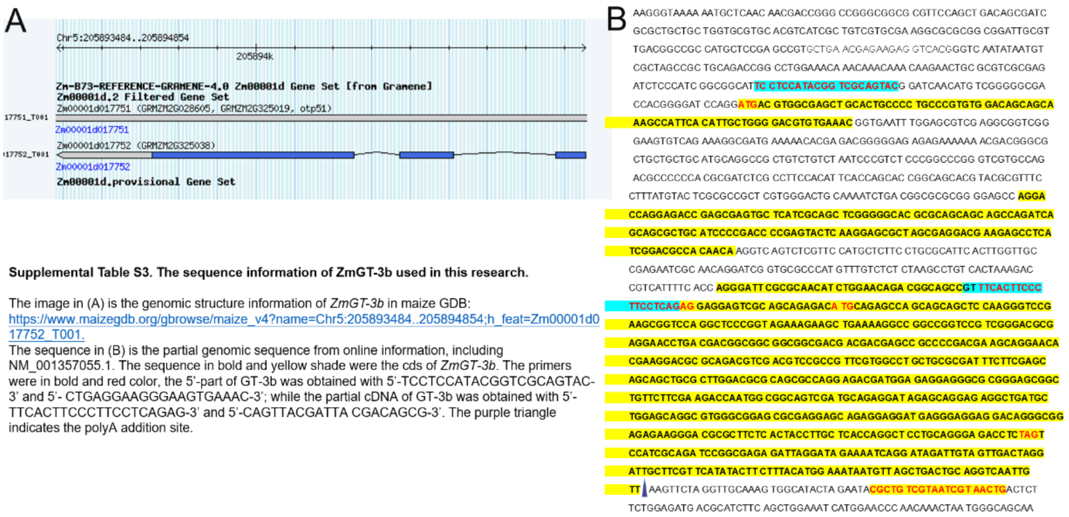

Table S3. The sequence information of ZmGT-3b used in this research. The image in (A) is the genomic structure information of *ZmGT-3b* in maize GDB: https://www.maizegdb.org/gbrowse/maize_v4?name=Chr5:205893484..205894854;h_feat=Zm00001d017752_T001. The sequence in (B) is the partial genomic sequence from online information, including NM_001357055.1. The sequence in bold and yellow shade were the cds of *ZmGT-3b*. The primers were in bold and red color, the 5'-part of GT-3b was obtained with 5'-TCCTCCATACGGTCGCAGTAC-3' and 5'- CTGAGGAAGGGAAGTGAAAC-3'; while the partial cDNA of GT-3b was obtained with 5'-TTCACTTCCCTTCCTCAGAG-3' and 5'-CAGTTACGATTACGACAGCG-3'. The purple triangle indicates the polyA addition site.
